# Rapid heterogeneous assembly of multiple magma reservoirs prior to Yellowstone supereruptions

**DOI:** 10.1038/srep14026

**Published:** 2015-09-10

**Authors:** Jörn-Frederik Wotzlaw, Ilya N. Bindeman, Richard A. Stern, Francois-Xavier D’Abzac, Urs Schaltegger

**Affiliations:** 1 Department of Earth Sciences, University of Geneva, Geneva, Switzerland; 2 Institute of Geochemistry and Petrology, Department of Earth Sciences, ETH Zurich, Switzerland; 3 Department of Geological Sciences, University of Oregon, Eugene, OR, USA; 4 Department of Earth and Atmospheric Sciences, University of Alberta, Edmonton, Canada; 5 Canadian Centre for Isotopic Microanalysis, University of Alberta, Edmonton, Canada

## Abstract

Large-volume caldera-forming eruptions of silicic magmas are an important feature of continental volcanism. The timescales and mechanisms of assembly of the magma reservoirs that feed such eruptions as well as the durations and physical conditions of upper-crustal storage remain highly debated topics in volcanology. Here we explore a comprehensive data set of isotopic (O, Hf) and chemical proxies in precisely U-Pb dated zircon crystals from all caldera-forming eruptions of Yellowstone supervolcano. Analysed zircons record rapid assembly of multiple magma reservoirs by repeated injections of isotopically heterogeneous magma batches and short pre-eruption storage times of 10^3^ to 10^4^ years. Decoupled oxygen-hafnium isotope systematics suggest a complex source for these magmas involving variable amounts of differentiated mantle-derived melt, Archean crust and hydrothermally altered shallow-crustal rocks. These data demonstrate that complex magma reservoirs with multiple sub-chambers are a common feature of rift- and hotspot related supervolcanoes. The short duration of reservoir assembly documents rapid crustal remelting and two to three orders of magnitude higher magma production rates beneath Yellowstone compared to continental arc volcanoes. The short pre-eruption storage times further suggest that the detection of voluminous reservoirs of eruptible magma beneath active supervolcanoes may only be possible prior to an impending eruption.

Supereruptions release hundreds to thousands of cubic kilometres of magma during single eruptions causing collapse of large calderas, formation of extensive pyroclastic flows and continent-wide ash fall deposits while releasing volcanic gases that have long-lasting impacts on global atmospheric circulation and climate^1^,^2^,^3^. The Yellowstone Plateau volcanic field in Idaho and Wyoming (USA) is arguably the most iconic active supervolcano on Earth. During the past ~2.1 million years it produced a cumulative volume of more than 4,500 km^3^ of rhyolitic lava flows and tuffs^4^,^5^. More than half of this volume was erupted during three of the largest-volume caldera-forming eruptions of the Pleistocene Epoch^4^. Voluminous lava flows as young as ~70,000 years, ongoing seismic and hydrothermal activity as well as geophysically imaged partial melt zones beneath Yellowstone caldera are evidence for an active upper-crustal magmatic-hydrothermal system^6^,^7^,^8^,^9^,^10^,^11^. Ultimately, this magmatic system is fuelled by an underlying mantle plume that provides heat through injections of basaltic magma into the overriding continental crust^12^,^13^.

Three major caldera forming eruptions are distinguished at Yellowstone and are represented by the ~2.1 Ma Huckleberry Ridge Tuff (HRT) with a total erupted volume of more than 2500 km^3^, the ~1.3 Ma Mesa Falls Tuff (MFT, 280 km^3^), and the 0.62 Ma Lava Creek Tuff (LCT, ~1000 km^3^) that resulted in collapse of the Yellowstone caldera. While MFT consists of a single ignimbrite flow unit, both HRT and LCT comprise three and two eruptive members, referred to as HRT-A, B and C as well as LCT-A and B, respectively^4^ (Fig. 1). The lack of significant stratigraphic gaps and soil formation between the distinct flow units suggest that the different members of HRT and LCT belong to the same respective cooling unit and were erupted in close succession. The areal distribution of the different members, however, suggest that they were erupted through distinct vents along their caldera margins^4^ (Fig. 1). The presence of amphibole in LCT-A and the large isotopic variations between different HRT members, particularly the unradiogenic Nd and radiogenic Sr and Pb isotopic signatures of HRT-C^14^,^15^, indicate that the different units tapped distinct reservoirs. Motivated by these petrologic and isotopic differences, previous ^40^Ar/^39^Ar geochronology studies suggest that LCT-B is significantly younger than LCT-A^16^ and HRT-C is distinguishably younger than the more voluminous HRT members A and B^17^, thus corresponding to distinct eruptions separated by several thousand years. Such a temporal dissection of the major Yellowstone ignimbrites into a series of less voluminous events would have significant implications for the frequency and magnitude of eruptions in the Yellowstone Plateau volcanic field, the Snake River Plain, and the frequency of supervolcanic events in general^18^,^19^.

Advanced *in-situ* geochemical methods coupled with high-precision geochronological techniques applied to the crystal cargo of ancient eruptions provide significant insights into the mechanisms and durations of magma accumulation prior to caldera-forming eruptions^20^,^21^,^22^,^23^,^24^. By applying these techniques to the same zircon crystals, time-resolved chemical and isotopic records can be constructed that permit a deciphering of the changing sources of individual magma batches, their lifetimes and chemical evolution prior to eruption. These records also allow predictions of the physical states of magma bodies in the upper crust and the mechanisms that trigger large-volume eruptions^19^,^21^,^22^,^25^. Here we present a comprehensive data set including *in situ* oxygen isotope and trace element analyses, hafnium isotope analyses and high-precision U-Pb geochronology, all performed on the same crystals of accessory zircon (see supplementary material for details) from the three caldera forming eruptions at Yellowstone. This data set provides a record of magma accumulation, magma sources, and pre-eruption evolution of the underlying magmatic systems at previously unattainable temporal resolution of 10^3^ to 10^4^ years. We show that the rapid assembly of multiple isotopically and chemically distinct shallow-crustal magma reservoirs is a common feature prior to Yellowstone supereruptions and likely characteristic of many other rift- and hotspot-related supervolcanoes on Earth.

## Results and Discussion

### Timing of magma accumulation and eruption at Yellowstone supervolcano

High-precision zircon U-Pb geochronology has been carried out for all major supereruptive units from Yellowstone, providing a precise and accurate eruption history for the voluminous tuffs, and also providing the crucial high-resolution temporal framework for the isotopic and chemical data from the same crystals. Ninety-three single zircon crystals or crystal fragments were dated employing chemical abrasion-isotope dilution-thermal ionization mass spectrometry techniques (Fig. 2; Table S1). We use these data to quantify reservoir assembly and storage time-scales and test the suggested temporal dissections of some of the major tuff units.

The first eruption of rhyolitic magma in the Yellowstone volcanic field produced the Snake River Butte (SRB) flow, a normal δ^18^O lava (Fig. 3), erupted from the south-western margin of what was to become the first-cycle caldera^4^. Nine out of twelve dated zircons from the SRB yielded indistinguishable initial ^230^Th-corrected (see supplementary material for details) ^206^Pb/^238^U dates with a weighted mean of 2.1506 ± 0.0056 Ma (MSWD = 0.79). Three crystals yielded distinguishably older dates between 2.198 ± 0.024 and 2.244 ± 0.028 Ma (Fig. 2). Despite excellent reproducibility between the two laboratories demonstrated in previous studies^26^, the weighted mean is 21 ± 10 ka younger than the youngest cluster of zircons dated by Rivera *et al.*^24^. However, the range in dates is similar in both data sets and we attribute this difference to real variations in the crystallization ages of dated zircons. These data suggest that the SRB erupted ~70 ka before the first caldera forming eruption of the HRT.

We dated zircons from all three members of the HRT. Seven of eight ^206^Pb/^238^U dates of HRT-A zircons are statistically equivalent with a weighted mean of 2.0798 ± 0.0094 Ma (MSWD = 0.86). The excluded single crystal date of 2.212 ± 0.069 Ma corresponds to a crystal with a distinct oscillatory zoned interior domain (Fig. S2) that has different trace element characteristics (Fig. 4) and is interpreted as an antecrystic core of SRB or similar origin. HRT-B yielded a more complex distribution of single crystal dates with a population of eleven statistically equivalent ^206^Pb/^238^U dates and a weighted mean of 2.0794 ± 0.0047 Ma (MSWD = 1.3). Four zircons yielded statistically distinguishable dates between 2.093 ± 0.010 to 2.178 ± 0.021 Ma, reflecting either recycling of antecrystic zircons or prolonged zircon crystallization during reservoir assembly. The distribution of our zircon dates is similar to those reported for HRT-B in previous studies^24^,^27^ and the weighted mean of the youngest statistically equivalent population in our data set is indistinguishable from the weighted mean U-Pb date of Singer *et al.*^27^ and slightly younger than but still overlapping within uncertainty with the U-Pb date of Rivera *et al.*^24^. Our U-Pb weighted mean U-Pb date for HRT-B is also in agreement with a consistent set of sanidine ^40^Ar/^39^Ar dates for the same unit^16^,^24^,^27^,^28^ (all relative to the calibration of ref. 29; Fig. 2). The short duration of zircon crystallization and the agreement with ^40^Ar/^39^Ar sanidine dates suggest that most analysed zircons crystallized close to eruption. These consistent data provide an excellent framework for our single crystal isotopic and chemical data and make HRT-B one of the best dated supereruptive units worldwide. The last eruption of the first Yellowstone cycle is represented by the HRT-C deposit. Eleven of twelve analyses of HRT-C zircons yielded indistinguishable ^206^Pb/^238^U dates with a weighted mean of 2.0783 ± 0.0083 Ma (MSWD = 1.3). One single analysis yielded a slightly older zircon crystallization age of 2.115 ± 0.034 Ma, equivalent to some of the older grains in HRT-B (Fig. 2). The short duration of zircon crystallization and the excellent agreement with a consistent set of sanidine ^40^Ar/^39^Ar dates suggest that the bulk of the eruption-feeding magma reservoirs were assembled within some 10^3^ to 10^4^ years before eruption. Even the oldest zircons from HRT and the majority of SRB zircons do not provide any evidence for a pre-eruption history longer than ~70 ka. The data further suggest that the HRT indeed represents one of the largest single eruptions of the Pliocene and not a series of distinct smaller-volume events (cf., ref. 17). This conclusion is in agreement with the consistent but unusual excursional paleomagnetic direction of all three HRT members that record an event of very short (few 10^3^ years) duration^27^,^30^.

The single member of MFT also yielded a simple distribution of zircon dates. Thirteen of fifteen analysed zircons yielded equivalent ^206^Pb/^238^U dates with a weighted mean of 1.3004 ± 0.0073 Ma (MSWD = 1.3) that is slightly younger but overlaps with previously reported sanidine ^40^Ar/^39^Ar dates of 1.313 ± 0.11 and 1.321 ± 0.024 Ma^16^,^28^ (Fig. 2). This suggests that the bulk of the erupted volume was assembled rapidly within the uncertainty of our geochronology in the shallow crust and that the majority of zircons, most of which have diverse low-δ^18^O signatures (Fig. 3), record melting-crystallization processes operating close to eruption.

Zircons from members A and B of LCT yielded simple distributions of ^206^Pb/^238^U dates. All analysed crystals from LCT-A yielded equivalent dates with a weighted mean of 0.6260 ± 0.0026 Ma (MSWD = 0.72) that is resolvably younger than previously reported sanidine ^40^Ar/^30^Ar dates of 0.655 ± 0.006 and 0.652 ± 0.012 Ma for the same unit^16^,^28^. Thirteen of fifteen LCT-B zircons yielded equivalent dates with a weighted mean of 0.6292 ± 0.0043 Ma (MSWD = 0.91) that is indistinguishable from the weighted mean of LCT-A zircons but resolvably different or barely overlapping with previous sanidine ^40^Ar/^39^Ar dates of 0.615 ± 0.008 Ma^16^ and 0.650 ± 0.016 to 0.649 ± 0.14 Ma^28^ (Fig. 2). Similarly to the HRT members, these data suggest that isotopically diverse zircons (Fig. 3) crystallized simultaneously and record rapid assembly of their host magma batches into voluminous reservoirs that fed the LCT eruptions. Taking the weighted mean zircon U-Pb dates as the best estimates for the eruption ages of LCT-A and LCT-B indicates that the two members were erupted very close in time and represent the same eruptive event.

Collectively, these data support previous claims of rapid magma assembly and short duration of pre-eruption shallow crustal magma storage beneath “hot and dry” Yellowstone-type supervolcanoes^22^,^24^ contrary to continental arc-type “cold and wet” large-volume silicic eruptions^21^,^31^,^32^,^33^. Our new data firmly establish this model for all supereruptive units of the Yellowstone Plateau volcanic field, consistent with its predecessor at the Heise volcanic field^22^. We now focus on remarkable oxygen and hafnium isotopic diversity within and between analysed zircon populations to delineate magma sources. We further use trace element characteristics of zircons to track down fractional crystallization trends that reflect the thermal evolution of the magma reservoirs preceding eruption.

### Oxygen and hafnium isotopic evidence for multiple magma reservoirs, mantle-crust interaction and shallow-crustal recycling

**Figure 1:**
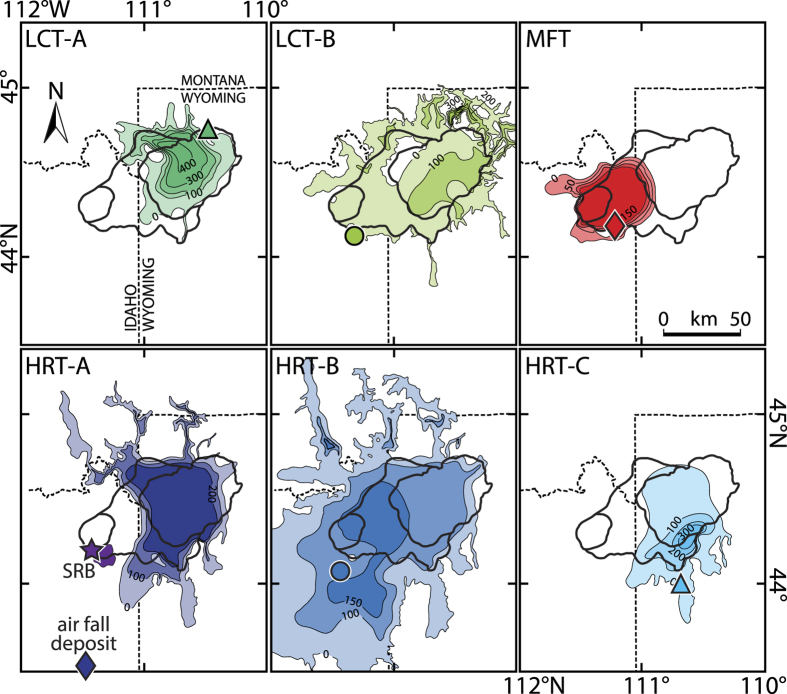
Caldera map and areal distributions of major caldera-forming eruptions at Yellowstone. The different panels show areal distributions of individual members of the three major Yellowstone eruptions with isopach contour lines from ref. 4. Labels on the contour lines denote the thickness in meters. Thick black lines are caldera margins^4^. Inferred locations of eruptive vents correspond to areas of greatest thickness of the corresponding deposit. Symbols indicate sample locations (for details see supplementary material).

**Figure 2:**
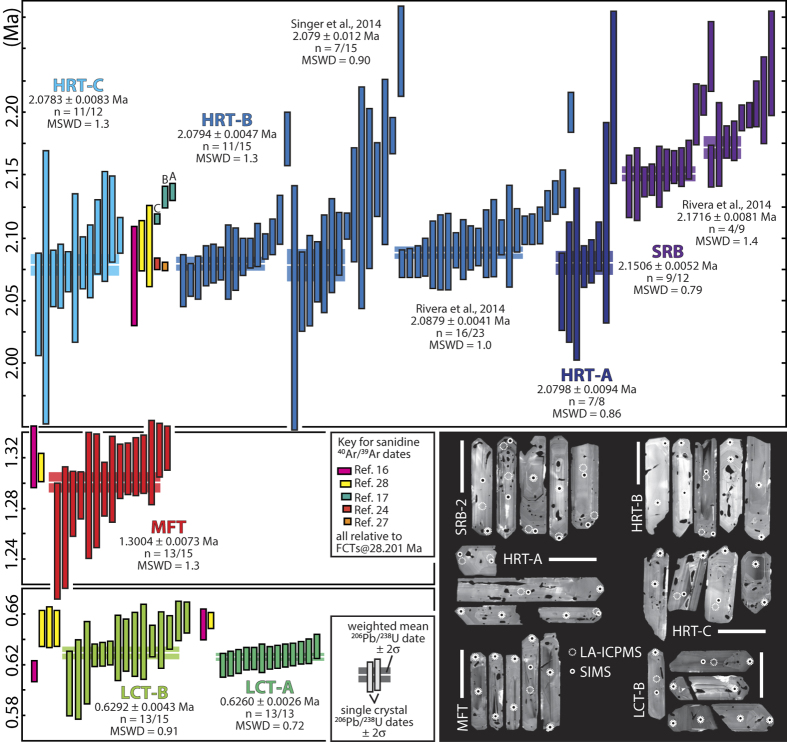
High-precision zircon U-Pb geochronology of Yellowstone supereruptions. Displayed are single crystal ^206^Pb/^238^U dates (vertical bars) and weighted mean dates of statistically equivalent clusters (horizontal bars). All ^206^Pb/^238^U dates were corrected for intitial ^238^U-^230^Th disequilibrium employing a constant Th-U partition coefficient ratio of 0.214 ± 0.079 based on published values and underpinned by new analyses of zircon-hosted melt inclusions from Yellowstone tuffs (see supplementary material for details). Previously published zircon U-Pb data^24^,^27^ were recalculated employing the same ^230^Th-disequilibrium correction. Also shown are selected published sanidine ^40^Ar/^39^Ar dates recalculated employing the calibration of ref. 29. Bottom right panel shows representative cathodoluminescence images of analysed zircons from all units. Circles represent locations of ion microprobe (SIMS) and laser ablation (LA-ICPMS) spots, respectively (see Fig. S1 for further details).

**Figure 3:**
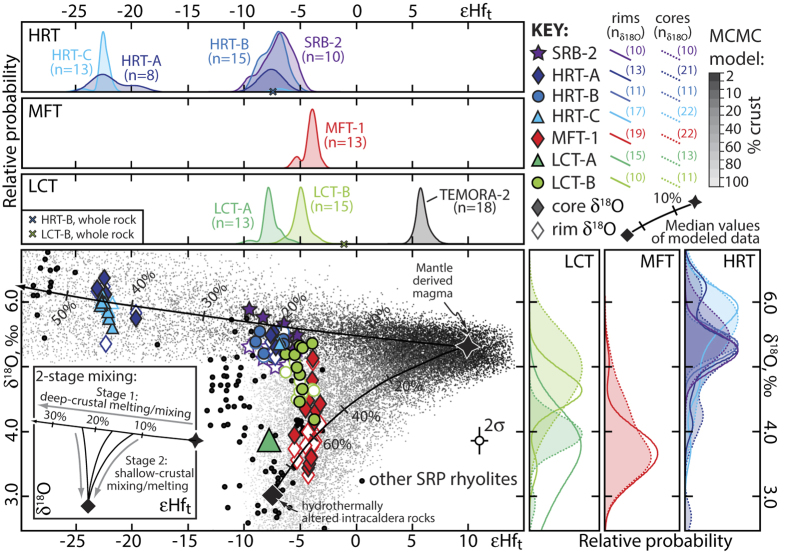
Oxygen and hafnium isotope systematics of Yellowstone zircons. Inter- and intracrystal oxygen and hafnium isotopic heterogeneities of Yellowstone zircons displayed as kernel density estimates and oxygen-hafnium isotope co-variance with Monte Carlo Markov Chain (MCMC) based model of mixing between mantle and crustal sources. The model calculates 10^3^ mixtures per model step (usually 10% steps with smaller 2% initial steps) for two-component mixtures of juvenile mantle melt (εHf = +5 to +15; 2–6 ppm Hf; δ^18^O = +5.0 to +5.6; refs. 34, 35, 36) and either Archean crust (εHf = −40 to −60; 4–8 ppm Hf; δ^18^O = +6 to +9; e.g., refs. 37,38) or variably hydrothermally altered low-δ^18^O HRT-equivalent intracaldera rocks (εHf = −5 to −10; 8–12 ppm Hf; δ^18^O = +1 to +5). Note that the kernel density estimates also include data from ref. 39 and hafnium data from crystals without oxygen isotope analyses. Also shown is the distribution of Hf isotope analyses of Temora-2 reference zircons for comparison (see supplementary material for details) and whole rock εHf for HRT-B and LCT-B reported by ref. 34. For LCT-A hafnium and oxygen isotopic data are from different crystals and the large symbol represents the respective median compositions of the populations.

The oxygen and hafnium isotopic compositions of Yellowstone zircons demonstrate the co-existence of multiple isotopically distinct eruption-feeding magma reservoirs at the resolution of our geochronology and their batch assembly histories in the shallow crust. Zircons from the SRB rhyolite and the three HRT members cluster in two distinct populations in hafnium-oxygen isotope space (Fig. 3). Zircons from HRT-A include both populations suggesting that at least two isotopically distinct reservoirs co-existed at the time of HRT-A eruption. These reservoirs either interacted before eruption or were mixed during simultaneous eruption from two vents. The isotopic compositions of the two HRT zircon populations are consistent with two component mixture models involving differentiated but isotopically juvenile mantle melts^34^,^35^,^36^ and Archean crustal melts^37^,^38^ at variable proportions (Fig. 3). Mixture modelling employing a Markov Chain Monte Carlo based approach suggests involvement of 15 to 25% and 40 to 50% of Archean crustal melt for the two reservoirs, respectively. HRT-B was exclusively derived from the reservoir with mantle-like oxygen isotopic composition and more juvenile hafnium in agreement with whole-rock Nd, Sr and Pb isotopic data^14^,^15^. SRB zircons isotopically largely resemble HRT-B zircons which is consistent with the close geographic relationship between the SRB lava flow and the HRT-B ignimbrite (Fig. 1) and geological observations by Christiansen^4^ that the SRB is a precursor unit closely related to HRT. Member C predominantly tapped the isotopically more crustal reservoir, also in agreement with bulk rock Nd isotopic data^15^ (Fig. 3).

HRT zircons within each of the two main clusters also display resolvable intercrystal hafnium isotopic heterogeneities (Fig. 3) suggesting that each of the two larger-scale reservoirs were rapidly assembled from smaller isotopically heterogeneous magma batches. However, the normal-to-high δ^18^O signature of the majority of these zircons and the lack of significant inter- and intracrystal oxygen isotopic variations suggest that assimilation of variable amounts of Archean crust occurred without recycling of low-δ^18^O hydrothermally altered material, and therefore likely deeper in the crust.

MFT zircons display inter- and intracrystal isotopic heterogeneity with respect to both oxygen and hafnium isotopes (Fig. 3). The low-δ^18^O signature of the majority of MFT zircons fingerprints shallow-crustal re-melting of meteoric-hydrothermally altered intracaldera rocks^20^,^22^,^23^,^37^,^39^,^40^. The relationship between the hafnium and oxygen isotopic compositions of MFT zircons is consistent with a two-component mixture between juvenile mantle melts and variably hydrothermally altered intracaldera rocks, likely consisting of altered and buried HRT-related rhyolites of similar composition and related intrusions as the HRT caldera almost entirely encloses the MFT deposit^4^ (Fig. 1). While the two-component mixture is consistent with the isotopic data, the magmas that were intruded into the shallow crust and that remelted and mixed with hydrothermally altered precursors, also likely ingested variable amounts of Archean crust during lower-to-mid crustal differentiation and ascent. We thus favour a 2-stage melting and mixing model involving deeper crustal melting of Archean crust that predominantly alters the hafnium isotopic composition followed by shallow-crustal remelting of altered intracaldera rocks causing a significant shift in oxygen isotopic composition (cf., refs. 15,36). This 2-stage process leads to a decoupling of oxygen and hafnium isotope systematics in zircon that is observed in our data and many other rhyolites from different caldera systems in the Snake River Plain (Fig. 3). Magma processing involving fractional crystallization and mixing with crustal melts at different crustal levels may be supported by recent seismic interpretations of a large-scale lower-crustal partial melt zone that is separated from the upper crustal reservoir previously imaged beneath Yellowstone (ref. 41).

Zircon populations from the two LCT members are distinct with respect to their oxygen and hafnium isotopic compositions (Fig. 3) suggesting that they tapped different magma reservoirs that were co-existing but physically separated prior to eruption. Inter- and intracrystalline isotopic heterogeneities in both populations support the heterogeneous batch assembly model involving 2-stage melting and sequestration of mantle derived magmas, Archean crust and shallow hydrothermally altered intracaldera rocks in variable proportions.

Conclusively, the paired single crystal oxygen and hafnium isotopic data highlight the importance of isotopic heterogeneities in tracing the assembly of upper-crustal supervolcanic magma reservoirs from various smaller-scale magma batches that are mixtures of multiple sources. These data also highlight the complexity of these eruption-feeding reservoirs, which comprise multiple co-existing but physically separated sub-chambers.

The inter- and intracrystalline zircon oxygen isotopic heterogeneities found in some of the studied Yellowstone units also provide independent mineral diffusive time scales for the duration of reservoir assembly and upper-crustal storage. Oxygen diffusion in zircon has been characterised experimentally for both dry and wet diffusion conditions by Watson and Cherniak^42^. While dry diffusion conditions are unrealistic for hydrous Yellowstone rhyolites, parameters for diffusion under wet conditions predict that diffusive oxygen isotopic equilibration in isotopically zoned zircons residing in hydrous rhyolitic magma at ~800 to 850 °C (see supplementary information for details on temperature estimates) requires several 10^3^ to 10^4^ years (see ref. 40 for further details). As oxygen isotopic heterogeneities are preserved, this provides maximum estimates for the pre-eruptive upper-crustal storage time of Yellowstone magmas. These maximum estimates are entirely consistent with the short duration of zircon crystallization derived from our geochronologic data.

### *In-situ* geochemical insights into the pre-eruption evolution of the shallow crustal magma reservoirs

**Figure 4:**
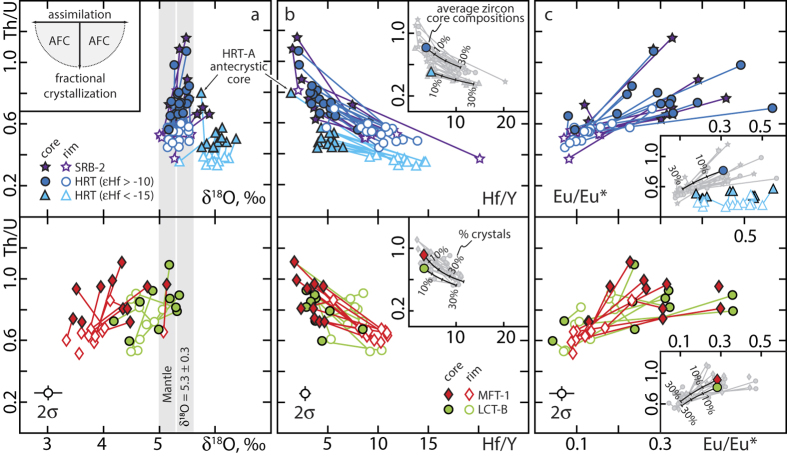
*In-situ* trace element and oxygen isotope variations in Yellowstone zircons. (**a**–**c**) Intra- and intercrystal variability and co-variance between Th/U and δ^18^O, Hf/Y and Eu/Eu* in core-rim pairs of Yellowstone zircons. Average 2σ uncertainties are based on the reproducibility of repeat analyses of reference zircons (see supplementary material for details). Th/U and Hf/Y are largely controlled by the fractionation of accessory allanite + chevkinite while Eu/Eu* is a signature of feldspar (sanidine + plagioclase) fractionation and δ^18^O is sensitive to shallow-crustal sequestration of hydrothermally altered intracaldera rocks. Insets in (**b**,**c**) show the same data (in grey) together with the results of modelling the impact of fractional crystallization on zircon trace element compositions. The model uses the average composition of zircon cores as the starting composition and computes changing zircon trace element compositions as a result of fractional crystallization (see supplementary material for further details). HRT zircon with low εHf do not show significant zoning with respect to Eu/Eu* and were not modelled but shown in the inset in panel (**c**) for comparison. AFC, assimilation-fractional crystallisation.

Microchemical analyses of oxygen isotopes and trace elements in zircon core-rim pairs record the processes operating during zircon crystallization and place even more detailed constraints on the pre-eruption assimilation and crystallization history^21^,^24^. Zircons from all units show systematic core-rim variations in trace element compositions that are consistent with cooling and co-crystallization of the mineral assemblage of the respective host tuff (Fig. 4).

Core-to-rim variations in trace element concentrations and ratios (e.g., Th/U, Hf/Y and Eu/Eu*; Fig. 4) of analysed HRT zircons are primarily controlled by crystallization of allanite + chevkinite and sanidine + plagioclase, respectively, and are entirely consistent with closed-system fractional crystallization (Fig. 4a–c). Grouping HRT zircons according to their hafnium isotopic composition instead of their host tuff clearly separates the two voluminous reservoirs and displays their distinct chemical evolutions within the two reservoirs prior to eruption (Fig. 4a–c). Pronounced Eu-anomalies in all HRT zircon cores likely reflect the derivation of juvenile and hot low-silica rhyolites by significant fractional crystallization of plume derived basalts at low oxygen fugacity prior to the onset of zircon crystallization. The majority of zircon rims have even more Eu-depleted compositions with Eu/Eu* as low as 0.1 (Fig. 4c), a signature of ongoing feldspar (sanidine + plagioclase) crystallization during zircon growth^24^. Exceptions are the HRT zircons with more crustal hafnium isotopes which possess rims that do not record the extremely low Eu/Eu* compared to zircons from the more juvenile reservoir. Zircons from this group also have lower Th/U at a given Hf/Y suggesting different trace element chemistry and fractional crystallization histories in the two reservoirs with lower overall crystal content and less feldspar crystallization in the more contaminated reservoir.

Zircons from MFT and LCT-B show core-rim trace element profiles similar to HRT units but additionally display systematic oxygen isotopic zoning, with the majority of crystals having rims that are more ^18^O-depleted than cores (Fig. 4a; Fig. S2). The co-variance between trace element and oxygen isotopic composition suggests progressive cannibalization of shallow-crustal rocks and fractional crystallisation during zircon growth.

Modelling the effect of fractional crystallization of typical Yellowstone mineral assemblages on the trace element composition of zircons shows that no more than 30% of crystallization is required to explain the observed core-to-rim variations (Fig. 4; see supplementary material). Considering that these tuffs have near-liquidus zircon saturation and Ti-in-zircon temperatures (for zircon cores; see supplementary material), this suggests that zircon trace element compositions do not record any evidence for prolonged storage at low temperature and high crystallinity and support the conclusions from our geochronologic and isotopic data for rapid assembly and short pre-eruption storage.

Although such rapid accumulation histories and short storage times for large-volume silicic magma reservoirs have been proposed previously^22^,^24^, numerous other studies prefer batholith-like accumulation of large volumes of magma stored as near-solidus crystal-rich mushes for >10^5^ years before eruption^21^,^33^,^43^,^44^,^45^,^46^. However, the hafnium and more significantly the oxygen isotopic diversity in zircon crystals of indistinguishable age found in most Yellowstone eruptive units suggests that large volumes of magma were assembled within 10^3^ to 10^4^ years, that is within the resolution of our high-precision U-Pb geochronology and at timescales too short for diffusive equilibration of oxygen isotopic heterogeneities in zircon. Our data further require complex sub-caldera reservoir configurations with multiple shallow magma reservoirs that were assembled from smaller isotopically heterogeneous magma batches. The short upper-crustal storage times of these complex reservoirs further suggest that the detection of such features beneath active Yellowstone-type supervolcanoes by geophysical techniques^6^,^41^,^47^,^48^ may only be possible within hundreds to thousands of years before an eruption.

## Methods summary

Zircons were separated from samples of all major eruptive units of the three supereruptive cycles of the Yellowstone volcanic field using conventional techniques. 30–50 zircons per sample were selected under a binocular microscope, placed in quartz crucibles and annealed at 900 °C for 48 hours. After annealing, zircons from SRB-2, HRT-A, B, C, MFT-1 and LCT-B were mounted in epoxy resin together with natural reference materials Mud Tank and Temora-2, ground and polished to expose crystal interiors and internal textures were characterised using back-scattered electron (BSE) and cathodoluminescence (CL) imaging employing a scanning electron microscope. Oxygen isotope analyses were performed at the Canadian Centre for Isotopic Microanalysis, University of Alberta, employing a Cameca IMS1280 secondary ion mass spectrometer similar to those reported in ref. 49. Repeat analyses of primary (Mud Tank zircon, δ^18^O_VSMOW_ = +4.87‰; R. Stern, unpublished data) reference zircons yielded within-session reproducibility between ±0.14 and 0.20‰ (2σ) that was propagated into the uncertainty of samples yielding an average single spot uncertainty of ±0.25‰. Analyses of secondary reference zircon Temora-2 (δ^18^O_VSMOW_ = +8.20‰; ref. 50) yielded a weighted mean δ^18^O_VSMOW_ of +8.260 ± 0.061‰ (MSWD = 1.6; n = 18). For samples, we preferentially targeted distinct core and rim domains identified in CL images to assess intracrystal isotopic zoning using a 15 μm probe diameter.

The same spots or domains were targeted for trace element analyses by laser ablation inductively coupled plasma mass spectrometry (LA-ICPMS) at the University of Lausanne employing a NewWave excimer laser ablation system attached to a Thermo ELEMENT2 ICP-MS and using NIST 612 glass as the primary reference material. Repeat analyses of Mud Tank zircon standard yielded external reproducibilities (2 R.S.D.) between 2.9% (Hf) and 18.1% (Ti). Additionally, exposed zircon-hosted melt inclusions (n = 5) were analysed for Th and U to quantify relative Th-U partitioning between zircon and melt. Analyses were performed with a 10 μm diameter laser beam and a fast scanning routine with static magnet to reduce settling times and reduce the depth of penetration of the laser.

After *in-situ* analyses, zircons were recovered from the epoxy mount, carefully cleaned in 3.5 N HNO_3_, transferred to 200 μl Savillex microcapsules and chemically abraded^51^ in concentrated HF for 13 hours at 180 °C. Chemically abraded zircons were cleaned, spiked with 3–5 mg of EARTHTIME ^202^Pb-^205^Pb-^233^U-^235^U tracer solution^52^ and dissolved in concentrated HF for 72 hours at 210 °C. After dissolution, U and Pb isotopes were separated from the matrix employing HCl-based ion exchange chromatography and analysed by thermal ionization mass spectrometry (Thermo TRITON) at the University of Geneva. Data reduction and uncertainty propagation were performed using U-Pb_Redux software^53^ that uses algorithms of ref. 54. The Zr-Hf and trace element fractions were collected during ion exchange chromatography and analysed for hafnium isotopes employing a Thermo NEPTUNE plus multi-collector ICPMS at the University of Geneva. Repeat analyses of Yb-doped and undoped JMC-475 reference material yielded an average of 0.282153 ± 0.000022 (2 S.D.), translating into an external reproducibility of ± 0.78 ε-units that was propagated into the uncertainties of samples and secondary reference materials. Analyses of secondary reference zircons Temora-2 and Plešovice yielded weighted mean εHf_t_ of 5.80 ± 0.20 (MSWD = 1.2; n = 18) and −3.33 ± 0.26 (MSWD = 1.3; n = 10), respectively.

Zircons from LCT-A and additional crystals from other units were only analysed for U-Pb and hafnium isotopes following the protocols outlined above. Details of the analytical protocols and mass spectrometry as well as full data tables and related additional figures are given in the supplementary material.

## Additional Information

**How to cite this article**: Wotzlaw, J.-F. *et al.* Rapid heterogeneous assembly of multiple magma reservoirs prior to Yellowstone supereruptions. *Sci. Rep.* **5**, 14026; doi: 10.1038/srep14026 (2015).

## Supplementary Material

Supplementary Information
