# Supplementary material for: Rapid heterogeneous assembly of multiple magma reservoirs prior to Yellowstone supereruptions
Source: Sci Rep. 2015 Sep 10;5:14026. doi: 10.1038/srep14026 (PMC4564848; doi:10.1038/srep14026)
Supplement: Supplementary Information [file srep14026-s1.pdf]

Supplementary Material

Rapid heterogeneous assembly of multiple magma reservoirs prior to Yellowstone supereruptions

Wotzlaw, J.F., Bindeman, I.N., Stern, R.A., D'Abzac, F.X., Schaltegger, U.

Content of supplementary material:

[1] Sample localities and description

[2] Analytical methods

[2.1] Oxygen isotope analysis
[2.2] Trace element analyses of zircons and melt inclusions
[2.3] Uranium-lead geochronology
[2.4] Hafnium isotope analysis
[2.5] Zircon trace element modelling

[3] Supplementary Figures and Data Tables

[4] Supplementary References

[1] Sample localities and description

SRB-2, Snake River Butte rhyolite, large volume pre-HRT lava flow, same sample as in Bindeman and Valley[55]. Sample locality: Snake River Butte, southern slope, **111°20'31" 44°07'51"**.

HRT-A, Huckleberry Ridge Tuff Member A, earliest HRT fall deposit, carefully picked individual pumice clasts sampled by I.N.B. in 2012. Sample locality: Ririe Reservoir Blacktail recreation area, **111°29'56.5" 43°29'56.5"**.

HRT-B, Huckleberry Ridge Tuff Member B, same sample as HRT-1 of Bindeman and Valley[55] and Bindeman et al.[40]. Previously dated by SHRIMP[56]. Sample locality: Upper Arcadia Reservoir, north of Ashton, **111°23'27" 44°05'55"**.

HRT-C, Huckleberry Ridge Tuff Member C, same sample as HRT-C of Bindeman and Valley[55] and Bindeman et al.[40]. Sample locality: Lizard Creek, Grand Teton National Park, **110°41'05" 44°00'25"**.

MFT-1, Mesa Falls Tuff, same sample as MFT-1 of Bindeman and Valley[55]and Bindeman et al.[40]. Sample locality: Upper Mesa Falls bluff, near the river, **111°13'38" 44°11'18"** (GPS coordinates pre 2000, accurate within ± 50 m).

LCT-A, Lava Creek Tuff Member A, same sample as LCT-3a of Bindeman and Valley[55] and Bindeman et al.[40]. Previously dated by SHRIMP[56]. Sample locality: Slightly welded upper Tuff at Gibbon River, **110°28'02" 44°43'12"**.

LCT-B, Lava Creek Tuff Member B, sampled by I.N.B. in 2012. Sample locality: Highway 20 between Island Park and Ashton, same outcrop as sample LCT-2 of Bindeman and Valley[55], **111°28'05" 44°16'00"**.

[2] Analytical techniques

For the majority of zircons analysed in this study we use analytical protocols that allow the analysis of oxygen isotopes, trace elements, U-Pb and Hf isotopes on the same crystals employing four different mass spectrometers. The entire sequence on analyses is illustrated in Fig. S1 and the protocols of individual analytical techniques is given in detail below.

[2.1] Oxygen isotope analysis

Sample preparation and secondary ion mass spectrometry (SIMS) were carried out at the Canadian Centre for Isotopic Microanalysis (CCIM), University of Alberta. Polished zircon mid-sections of unknowns and zircon reference materials were exposed within a 25 mm diameter epoxy mount (M1174) using diamond grits. The mount was cleaned with a lab soap solution, and de-ionized H_2O. Prior to scanning electron microscopy (SEM), the mounts was coated with 5 nm of high-purity Au. SEM characterization was carried out with a Zeiss EVO MA15 instrument equipped with a high-sensitivity, broadband cathodoluminescence (CL) detector and a backscattered electron detector. Typical beam conditions were 15 kV and 3–4 nA. A further 25 nm of Au was subsequently deposited on the mount prior to SIMS analysis. CL images of all zircons are given in Fig. S2.

Oxygen isotopes (^{18}O, ^{16}O) in zircon were analyzed using a Cameca IMS 1280 multicollector ion microprobe. A $^{133}Cs+$ primary beam was operated with impact energy of 20 keV and beam current of ~ 3.0 nA. The ~12 µm diameter probe was rastered (15 x 15 µm) for 30 s prior to acquisition, and then 5 x 5 µm during acquisition, forming rectangular analyzed areas ~15 x 18 µm across and ~2 µm deep. The normal incidence electron gun was utilized for charge compensation. Negative secondary ions were extracted through 10 kV into the secondary (Transfer) column. Transfer conditions included a 122 µm entrance slit, a 5 x 5 mm pre-ESA (field) aperture, and 100x sample magnification at the field aperture, transmitting all regions of the sputtered area. No energy filtering was employed. The mass/charge separated oxygen ions were detected simultaneously in Faraday cups L'2 (^{16}O-) and H1 (^{18}O-) at mass resolutions (m/Δm at 10%) of 1900 and 2250, respectively. Secondary ion count rates for ^{16}O- and ^{18}O- were typically ~3 x 10^9 and 6 x 10^6 counts/s utilizing 10^{10} Ω and 10^{11} Ω amplifier circuits, respectively. Faraday cup baselines were measured at the start of the analytical session. A single analysis took 4 minutes, including pre-analysis rastering, automated secondary ion tuning, and 90 s of continuous peak counting.

Instrumental mass fractionation (IMF) was monitored by repeated analysis of a zircon primary reference material (RM), S0081 (UAMT1) with $\delta^{18}O_{VSMOW}$ = +4.87 (R. Stern, unpublished laser fluorination data from Ilya Bindeman, University of Oregon) and a secondary zircon RM, S0022 (Temora-2) zircon with $\delta^{18}O_{VSMOW}$ = +8.20 ‰ (ref. 49). One analysis of the primary and secondary RM was taken after every 4 and 8 unknowns, respectively. The data set of ^{18}O-/^{16}O- for S0081 zircon was processed collectively for each of four analytical sessions, yielding a standard deviation of 0.07 – 0.10 ‰ after corrrection for systematic within-session drift (<0.3 ‰). Overall correction for IMF was <0.3 ‰. The individual spot uncertainties at 95% confidence for $\delta^{18}O_{VSMOW}$ reported include errors relating to within-spot counting statistics, between-spot (geometric) effects, and correction for instrumental mass fractionation, and average ±0.25 ‰ (Tab. S1). Accuracy and reproducibility of these protocols was assessed by repeat analyses of secondary reference zircon Temora-2 (S0022); twenty-six analyses, processed as unknowns, yielded a mean $\delta^{18}O_{VSMOW}$ = +8.260 ± 0.061 (MSWD = 1.6, n = 26; Fig. S3).

[2.2] Trace element analyses of zircons and melt inclusions

Trace elements in zircons were measured by laser ablation inductively coupled plasma mass spectrometry (LA-ICPMS) at the University of Lausanne employing a NewWave UP-193FX ArF excimer laser ablation system coupled to a Thermo Element XR sector-field ICPMS. The laser ablation system was operated with a repetition rate of 5 Hz, a spot diameter of 25 µm and an on-sample energy density of 3 J/cm^2. The glass standard reference material (SRM) 612 of the National Institute of Science and Technology (NIST) was used for external standardization and all intensities were internally normalized to ^{29}Si. During data reduction, care was taken to avoid parts of the time-resolved signals that were affected by ablation of mineral or melt inclusions. In some cases not all of these signal parts could be avoided leading to elevated non-stoichiometric bivalent elements as well as elevated LREE (mainly related to feldspar and/or apatite inclusions). Repeat analyses of secondary zircon reference material Mud

Tank yielded external reproducibilities (2 R.S.D.) between 2.9 % for Hf and 18.1 % for Ti (Tab. S1; Fig. S3). Temora-2 is heterogeneous with respect to most trace elements but the analyses are in good agreement with previously reported data (Ref. 49). Mud Tank is homogeneous but has significantly lower trace element concentrations compared to our samples, suggesting that the reproducibility can be considered a maximum uncertainty for the samples.

Th/U (^{238}U and ^{232}Th) was measured in zircon-hosted melt inclusions of some of the analysed crystals (n=5; Tab. S1; Fig. S5) using the same instruments as for zircons. Due to the small size of the analysed melt inclusions, the ablation system was operated with a pit diameter of 10 µm at a repetition rate of 7 Hz. We employed a fast scanning routine with static magnet to reduce magnet settling times and depth penetration. Mass bias was quantified using repeat analyses of NIST SRM 612. Time-resolved signals were carefully evaluated to avoid signal parts affected by ablation of host-zircon material. Th/U was calculated from $^{232}Th/^{238}U$ assuming a $^{238}U/^{235}U$ of 137.8.

[2.3] Uranium-lead geochronology

Following in situ oxygen isotope and trace element analyses, zircons were recovered from the grain mounts and analysed for U-Pb isotopes employing chemical abrasion isotope dilution thermal ionization mass spectrometry (CA-ID-TIMS) techniques at the University of Geneva following established procedures (Refs. 21, 22, 57). All crystals were annealed by heating to 900°C for 48 h in a muffle furnace prior to in-situ analyses. Individual crystals were subsequently extracted from the grain mounts under a binocular microscope, transferred into 3 ml Savillex beakers, rinsed with 3 N HNO_3 and loaded into 200 µl Savillex microcapsules for partial dissolution (i.e., "chemically abraded"; ref. 51) in HF + trace HNO_3 at 180°C for 15 h in Parr bombs. After partial dissolution, residual crystals/crystal fragments were transferred back into 3 ml Savillex beakers, rinsed with water, fluxed for several hours in 6 N HCl and ultrasonically cleaned in 3 N HNO_3. Zircons were then reloaded into their respective 200 µl microcapsules, spiked with 3-5 mg of the EARTHTIME ^{202}Pb-^{205}Pb-^{233}U-^{235}U tracer solution (hereafter referred to as ET2535; tracer calibration v. 3.0; http://www.earth-time.org/; ref. 52) and dissolved in ~70 µl HF at 210°C for >72 h in Parr bombs. After dissolution, samples were dried down and redissolved in 6 N HCl at 180°C overnight, dried down again and redissolved in 3 N HCl. U and Pb were then separated using a modified HCl-based single-column anion exchange chemistry[58]. The U-Pb fractions were dried down with a microdrop of 0.02 M H_3PO_4 and loaded on outgassed single Re-filaments with a Si-Gel emitter[59]. U and Pb isotopic measurements were performed on a Thermo TRITON thermal ionization mass spectrometer. Pb was measured in dynamic mode on a MasCom secondary electron multiplier. Analyses of ET2535-spiked samples were corrected for instrumental mass fractionation using the fractionation factor derived from the measured $^{202}Pb/^{205}Pb$ ratio relative to a true value of 0.99924. U was measured as U-oxide in static mode on Faraday cups equipped with 10^{12} Ω resistors. Measured isotopic ratios were corrected for isobaric interferences of $^{233}U^{18}O^{16}O$ on $^{235}U^{16}O_2$ using an $^{18}O/^{16}O$ of 0.00205 and for mass fractionation using the measured $^{233}U/^{235}U$ ratio relative to a value of 0.99506 for both tracers and a sample $^{238}U/^{235}U$ of 137.818 ± 0.045 (2σ; ref. 60). Total procedural blanks measured during the same period as the samples averaged 0.17 ± 0.09 pg (n = 17) and the average blank isotopic composition (see Tab. S2) was used for blank correction of zircon analyses. Common Pb in excess of blank was corrected using an estimate computed using the two-stage Pb evolution model of Stacey and Kramers[61]. Note that the computed common Pb composition is well within uncertainty of the measured blank composition. Data reduction was performed using Tripoli and U-Pb_Redux software[53] that employs algorithms of McLean et al.[54]. U-Pb ratios and dates were calculated relative to a $^{235}U/^{205}Pb$ ratio of 100.23 ± 0.046 % (2σ) and using the decay constants of ref. 62.

All $^{206}Pb/^{238}U$ dates were corrected for initial ^{238}U-^{230}Th disequilibrium in the ^{238}U-^{206}Pb decay chain that arises from preferential exclusion of Th relative to U during zircon crystallization (e.g., ref. 63). We here use a model correction assuming that variations in Th/U of analyzed zircons are due to variations in the Th/U of the magma in equilibrium with the respective zircon during crystallization and not due to variations in relative partitioning between Th and U (i.e., D_{Th}/D_U remains constant). We use a partition coefficient ratio D_{Th}/D_U derived from published empirical and experminetal data[64-68] and our analyses of melt inclusion-zircon pairs (Fig. S5). The average D_{Th}/D_U derived from our melt inlusion-zircon pairs is 0.214 ± 0.094 (Fig. S5). Including the published data yields an average of 0.214 ± 0.074 (Fig. S5), that we use for all analyses of zircons in this study and for recalculation of published data[24,27]. This correction results in a constant increase of $^{206}Pb/^{238}U$ dates by 86.5 ± 8.6 ka. Uncertainties associated with this correction were propagated into the uncertainty of $^{206}Pb/^{238}U$ dates of individual zircons.

All uncertainties are reported at the 95% confidence level. Single crystal dates are reported and plotted only with their analytical uncertainties excluding systematic contributions. However, for accurate comparison with $^{40}Ar/^{39}Ar$ dates, systematic uncertainties associated with the tracer isotopic composition and the ^{238}U decay constant have to be taken into account.

[2.4] Hafnium isotope analysis

Hafnium isotope analyses were conducted at the University of Geneva employing a THERMO Neptune Plus MC-ICP-MS equipped with nickel cones (Thermo X-series). Analyses were performed under dry plasma conditions employing a Cetac Aridus-2 desolvation unit and a Teflon nebulizer (~50µl /min uptake rate). The 9 Faraday cups were configured to measure the following masses at low mass resolution (m/Δm ~ 450): 172 (L4), 173 (L3), 175 (L2), 176 (L1), 177 (C), 178 (H1), 179 (H2), 180 (H3), 181 (H4). All analytes were diluted in 2 % HNO3 with traces of HF in order to minimize memory effects during analyses and stabilize Hf ions in solution. The certified JMC475 standard was used as a reference throughout the sessions, and was run at a concentration of ~30 ppb. A series of JMC475 solutions doped with variable amounts of Yb with concentrations ranging from 0.5 to 5 ppb (i.e., $0.001<{}^{173}Yb/{}^{177}Hf<0.5$) was prepared in order to optimize the ^{176}Yb interference correction during post-measurement data processing. Natural zircon standards Temora-2[50] and Plešovice[69] were measured as secondary reference materials. Two aliquots of Plešovice were also doped with Yb ($^{173}Yb/^{177}Hf \sim 0.23$). Samples were prepared from the Zr-Hf-trace element fractions of U-Pb column chemistry that were dried down and brought into solution using ~500 µl of the same batch of 2% HNO_3 used for analytes dilution and wash cycles (~400 s between analytes). Measurements included an 80-100 s uptake, baseline control over 30 s and 330 s (80 cycles of 4.2 s) of analysis, for a total consumption of ~450 µl. The βYb and βHf mass bias coefficients were calculated using an exponential law[70] from the measured $^{172}Yb/^{173}Yb$ and $^{179}Hf/^{177}Hf$ respectively and using natural abundances reference values: $^{172}Yb/^{173}Yb = 1.3534$ and $^{179}Hf/^{177}Hf = 0.7325$ (ref. 71). Mass bias correction on Lu was calculated using βYb and the isobaric interference of ^{176}Lu was removed using the natural abundances of ^{175}Lu (0.97416) and ^{176}Lu (0.02584). The isobaric interference of ^{176}Yb is the most significant and was evaluated from Yb doped JMC475 solutions by calculating $^{176}Yb/^{173}Yb$ using βYb and using a reference value that is empirically determined for each run by minimizing the dependence of corrected $^{176}Hf/^{177}Hf$ ratios on the measured $^{173}Yb/^{177}Yb$ (Fig. S3; ref. 72). Correction for in situ ^{176}Hf in-growth due to ^{176}Lu λ-decay has been calculated using $\lambda^{176}Lu$ of Scherer et al.[73] and the $^{206}Pb/^{238}U$ date of the respective zircon. The $^{176}Hf/^{177}Hf$ ratio for all JMC475 over the whole campaign of measurements is 0.282152 ± 22 (2 S.D., n=35), corresponding to a reproducibility of ± 0.78 ε-units (Fig. S4). All analyses were normalized to the recommended value of 0.282160 (ref. 74). Reported uncertainties of samples and secondary reference zircons include the within-run precision and the reproducibility of the JMC475 measurements propagated by quadratic addition. Accuracy and reproducibility of these protocols were assessed by repeat analyses of secondary zircon standards Plešovice and Temora-2 (Fig. S4). Analyses of Plešovice zircon solutions yielded a weighted mean εHf_t of -3.33 ± 0.26 (2σ; MSWD = 1.3; n = 10; t = 338 Ma) and repeat analyses of Temora-2 yielded a weighted mean εHf_t of 5.90 ± 0.20 (2σ; MSWD = 1.2; n = 18; t = 417 Ma) that are both in good agreement with previously reported data[69,75] (Fig. S4). All compositional and isotopic data for samples and reference materials are given in Tab S2.

[2.5] Zircon trace element modelling

We modelled the effect of fractional crystallization on zircon trace element compositions employing a simple mass balance model. The model uses the average compositon of zircon cores as the starting composition and models changing zircon trace element compositions as a consenquence of co-crystallization of a typical Yellowstone mineral assemblage. The model was run as a Markov Chain Monte Carlo simulation that randomly varies the input parameters within the specific limits with 10^4 simulations per step (10% steps; displayed are only median values of modelled populations). The fractionating assemblage of the model includes: Quartz (30-40%), sanidine (20-30%), plagioclase (20-30%), clinopyroxene (5-8%) and accessory zircon (0.01-0.04%), allanite and chevkinite (treated together; 0.02-0.2%). Partition coefficients for all modelled mineral phases were varied widely covering the full range of values of refs. 76-82.

[3] Supplementary Figures and Data Tables

Figure S1. Illustration of our analytical protocol that permits analyses of oxygen isotopes and trace elements in-situ prior to zircon dissolution followed by U-Pb and Hf isotope analyses on the same volume of dissolved zircons after ion exchange chemistry (modified from ref. 83). Oxygen isotope and trace element analyses were performed on the exact same location within a given crystal or within the same textural domains of zircons.

Figure S2. Cathodoluminescence images of analysed zircons from all studied Yellowstone units. Indicated are the locations of SIMS and LA-ICPMS spots. In the majority of cases laser ablation ICPMS analyses were performed on top of SIMS oxygen spots. In case of the presence of visible inclusions in the close vicinity or underneath the SIMS spots, LA-ICPMS analyses were performed at different location but within the same CL domain. Yellow numbers are $\delta^{18}O$ of the respective SIMS analysis. Details of melt inclusion analyses are given in Fig. S5.

Figure S3. Assessment of accuracy and external reproducibility of SIMS oxygen isotope and LA-ICPMS trace element analyses. (A) Accuracy and reproducibility of oxygen isotope analyses on secondary zircon standard Temora-2 (ref. 50). (B, C) Representative cathodoluminescence images of Mud Tank and Temora-2 zircon standards. (D) Reproducibility of trace element concentration and ratio determinations by LA-ICPMS on Mud Tank and Temora zircon standards. Note that Temora-2 is significantly more heterogeneous. The reproducibility of trace element analyses on Mud Tank zircon gives a maximum uncertainty estimate for unknowns when taking into account that concentrations for most elements are significantly lower in the standard compared to the unknowns.

Figure S4. Assessment of accuarcy and reproducibility of hafnium isotope analyses by solution nebulisation MC-ICPMS. (A) Reproducibility of primary reference material JMC-475. Repeat analyses yielded a reproducibility of 0.78 ε-units that was propagated into uncertainties of secondary reference zircons and unknowns. (B, C) Accuracy and reproducibility of secondary reference zircons Temora-2 and Plešovice. (D) Difference between measured values and reference values as a function of $^{173}Yb/^{177}Hf$ for all analysed reference materials demonstrating accuracy of the applied ^{176}Yb-interference correction[72].

Figure S5. Analyses of Th/U in Yellowstone zircon-hosted melt inclusions and the relative partitioning of Th and U between zircon and melt. Shown are a compilation of empirical and experimental estimates for the zircon-melt partition coefficient ratio of Th and U ($D_{Th/U}$) and $D_{Th/U}$ derived from our analyses of melt inclusion-zircon pairs from Yellowstone tuffs.

Figure S6. Details of oxygen isotope systematics of Yellowstone zircons. Shown are core and rim analyses from this study and ref. 40 displaying the level of inter- and intracrystal heterogeneity within individual samples.

Figure S7. Details of hafnium isotope systematics of Yellowstone zircons. (A) Single-crystal hafnium isotopic data for Yellowstone zircons with reduced chi-squared statistics (MSWD) to assess hafnium isotopic heterogeneities within samples. Also shown are the data for secondary reference zircons for direct comparison. Note the significantly larger scatter in the samples compared to the secondary standards, suggesting real isotopic diversity in the samples.

Figure S8. Comparison of temperature estimates for Yellowstone tuffs. Shown are liquidus temperatures and zircon saturation temperatures[55] recalculated using the calibration of Boehnke et al.[84] in comparison to median Ti-in-zircon temperatures of zircon cores and rims. Ti-in-zircon temperatures were calculated using the calibration of Ferry and Watson[85] with a_{SiO2}=1 and a_{TiO2}=0.55.

Table S1. In-situ oxygen isotopic (SIMS) and trace element (LA-ICPMS) data for analysed Yellowstone zircons, secondary zircon reference materials, and Th/U of zircon-hosted melt inclusions with calculated $D_{Th/U}$.

Table S2. U-Pb and Lu-Hf isotopic data for Yellowstone zircons and reference materials.

[4] Supplementary References

55. Bindeman, I.N. & Valley, J.W Low-δ^{18}O rhyolites from Yellowstone: Magmatic evolution based on analyses of zircons and individual phenocrysts. J. Petrol. 42, 1591–1517 (2001).
56. Bindeman, I.N. et al. Post-caldera volcanism: in situ measurement of U-Pb age and oxygen isotope ratio in Pleistcene zircons from Yellowstone caldera. Earth Planet. Sci. Lett. 189, 197–206 (2001).
57. Wotzlaw, J.F. et al. High-precision zircon U–Pbgeochronology of astronomically dated volcanic ash beds from the Mediterranean Miocene. Earth Planet. Sci. Lett. 407, 19–34 (2014).
58. Krogh, T.E. A low contamination method for hydrothermal decomposition of zircon and extraction of U and Pb for isotopic age determination. Geochim. Cosmochim. Acta 37, 485–494 (1973).
59. Gerstenberger, H. & Haase, G. A highly effective emitter substance for mass spectrometric Pb isotope ratio determinations. Chem. Geol. 136, 309–312 (1997).
60. Hiess, J. et al. ^{238}U/^{235}U systematics in terrestrial uranium-bearing minerals. Science 335, 1610–1614 (2012).
61. Stacey, J.S. & Kramers, J.D. Approximation of Terrestrial Lead Isotope Evolution by a 2-Stage Model. Earth Planet. Sci. Lett. 26, 207–221 (1975).
62. Jaffey, A.H. et al. Precision measurement of half-lives and specific activities of ^{235}U and ^{238}U. Phys. Rev., C4 (5), 1889–1906 (1971).
63. Schärer, U. The effect of initial ^{230}Th disequilibrium on young U-Pb ages: the Makalu case, Himalaya. Earth. Planet. Sci. Lett. 67, 191–204 (1984).
64. Fukuoka T. & Kagoshi K. Discordant Io-ages and the uranium and thorium distribution between zircon and host rocks. Geochem. J. 8, 117–122 (1974).
65. Blundy, J. & Wood, B. Mineral-melt partitioning of uranium, thorium and their daughters. Rev. Mineral. Geochem. 52, 59–123 (2003).
66. Bindeman, I.N., Schmitt, A.K. & Valley, J.W. U-Pb zircon geochronology of silicic tuffs from the Timber Mountain/Oasis Valley caldera complex, Nevada: rapid generation of large volume magmas by shallow-level remelting. Contrib. Mineral. Petrol. 152, 649–665 (2006).
67. Rubatto, D. & Hermann, J. Experimental zircon/melt and zircon/garnet trace element partitioning and implications for the geochronology of crustal rocks. Chem. Geol. 241, 38–61 (2007).
68. Guillong M. et al. LA-ICP-MS Pb–U dating of young zircons from the Kos–Nisyros volcanic centre, SE Aegean arc. J. Anal. At. Spectrom. 29, 963–970 (2014).
69. Sláma, J. et al. Plešovice zircon – A new natural reference material for U–Pb and Hf isotopic microana lysis. Chem. Geol. 249, 1–35 (2008).
70. Albarede, F. et al. Precise and accurate isotopic measurements using multiple-collector ICPMS. Geochim. Cosmochim. Acta 68, 2725–2744 (2004).
71. Blichert-Toft, J. & Albarède, F. The Lu-Hf isotope geochemistry of chondrites and the evolution of the mantle-crust system. Earth Planet. Sci. Lett. 148, 243–258 (1997).
72. Fisher, C. M., Vervoort, J.D. & Hanchar, J.M. Guidelines for reporting zircon Hf isotopic data by LA-MC-ICPMS and potential pitfalls in the interpretation of these data. Chem. Geol. 363, 125–133 (2014).
73. Scherer, E., Münker, C. & Mezger, K. Calibration of the Lutetium-Hafnium clock. Science 293, 683–687 (2001).
74. Vervoort, J. D. & Blichert-Toft, J. Evolution of the depleted mantle: Hf isotope evidence from juvenile rocks through time. Geochim. Cosmochim. Acta 63, 533–556 (1999).
75. Wu, F.-Y. et al. Hf isotopic compositions of the standard zircons and baddeleyites used in U–Pb geochronology. Chem. Geol. 234 105–126 (2006).
76. Schnetzler, C.C. & Philpotts, J.A., Partition coefficients of rare-earth elements between igneous matrix material and rock-forming mineral phenocrysts-II. Geochim. Cosmochim. Acta 34, 331-340 (1970).
77. Mahood, G. & Hildreth, W., Large partition coefficients for trace elements in high-silica rhyolites. Geochim. Cosmochim. Acta 47, 11-30 (1983).
78. Leeman, W.P. & Phelps, D.W. Partitioning of rare earths and other trace elements between sanidine and coexisting volcanic glass. J. Geophys. Res. 86, 10193-10199 (1981).
79. Nash, W.P. & Crecraft, H.R. Partition coefficients for trace elements in silicic magmas. Geochim. Cosmochim. Acta 49, 2309-2322 (1985).

80. Troll, V.R. et al. The REE-Ti mineral chevkinite in comendite magmas from Gran Canaria, Spain: a SYXRF-probe study. Contrib. Mineral. Petrol. 145, 730-741 (2003).
81. Bachmann, O., Dungan, M.A. & Bussy, F. Insights into shallow magmatic processes in large silicic magma bodies: The trace element record in the Fish Canyon magma body, Colorado. Contrib. Mineral. Petrol. 149, 338-349 (2003).
82. Olin, P.H. & Wolff, J.A. Rare earth and high field strength element partitioning between iron-rich clinopyroxenes and felsic liquids. Contrib. Mineral. Petrol. 160, 761-775 (2010).
83. Schoene, B. et al. A new method integrating high-precision U-Pb geochronology with zircon trace element analysis (U-Pb TIMS-TEA): Geochim. Cosmochim. Acta 74, 7144–7159 (2010).
84. Boehnke, P. et al. Zircon saturation re-visited. Chem. Geol. 352, 324-334 (2013).
85. Ferry, J.M. & Watson, E.B. New thermodynamic models and revised calibrations for the Ti-in-zircon and Zr-in-rutile thermometers. Contrib. Mineral. Petrol. 154, 429–437 (2007).

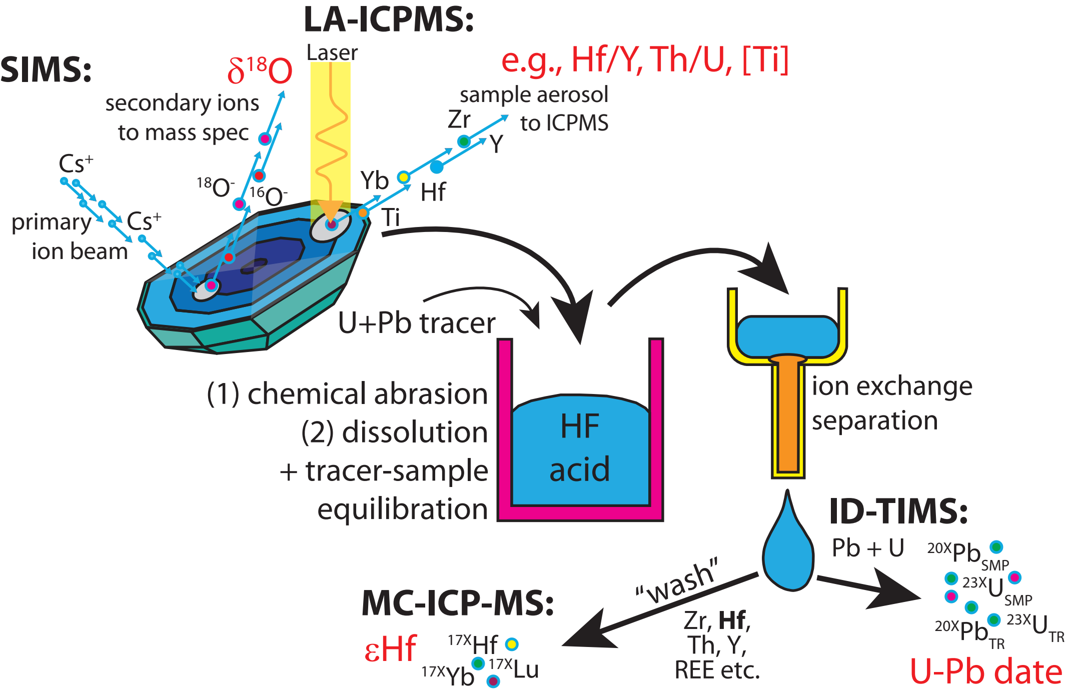

LA-ICPMS:
SIMS:
Laser
δ18O
e.g., Hf/Y, Th/U, [Ti]
secondary ions
to mass spec
sample aerosol
to ICPMS
Zr
Y
Cs+
Yb
Hf
18O-
16O-
Ti
primary
ion beam
Cs+
U+Pb tracer
ion exchange
separation
(1) chemical abrasion
(2) dissolution
+ tracer-sample
equilibration
HF
acid
ID-TIMS:
Pb + U
20XPbSMP
23XUSMP
20XPbTR
23XUTR
"wash"
MC-ICP-MS:
εHf
17XHf
17XYb
17XLu
Zr, Hf,
Th, Y,
REE etc.
U-Pb date

Figure S1

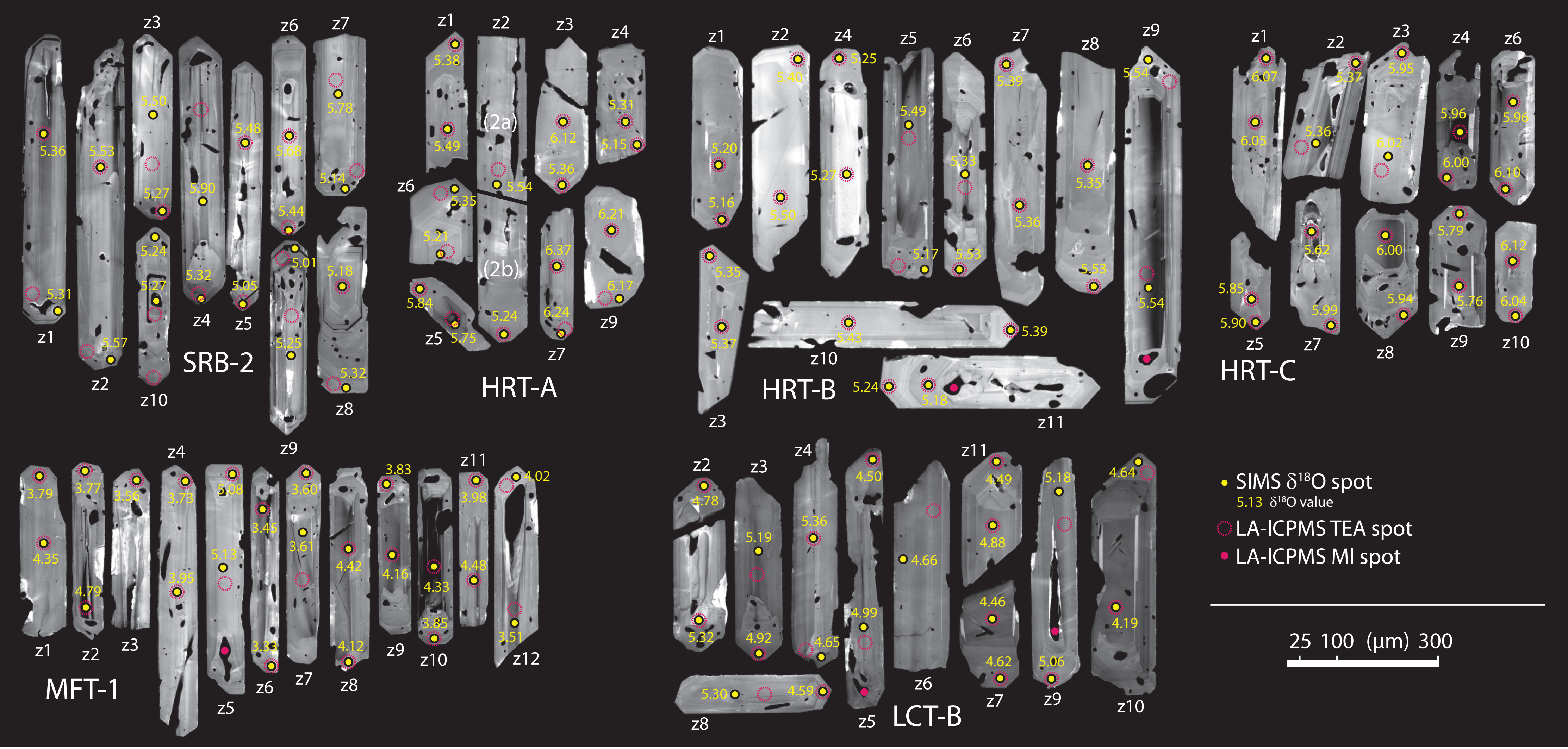

SRB-2
z1
5.36
5.31
z2
5.53
5.57
z3
5.50
5.27
z10
5.24
5.27
z4
5.90
5.32
z5
5.48
5.05
z6
5.68
5.44
z9
5.01
5.25
z7
5.78
5.14
z8
5.18
5.32
HRT-A
z1
5.38
5.49
z2
(2a)
5.54
(2b)
5.24
z3
6.12
5.36
z4
5.31
5.15
z6
5.35
5.21
z5
5.84
5.75
z7
6.37
6.24
z9
6.21
6.17
HRT-B
z1
5.20
5.16
z2
5.40
5.50
z4
5.25
5.27
z5
5.49
5.17
z6
5.33
5.53
z7
5.39
5.36
z8
5.35
5.53
z9
5.54
5.54
z3
5.35
5.37
z10
5.43
5.39
z11
5.24
5.18
HRT-C
z1
6.07
6.05
z2
5.37
5.36
z3
5.95
6.02
z4
5.96
6.00
z6
5.96
6.10
z5
5.85
5.90
z7
5.62
5.99
z8
6.00
z9
5.79
5.94
5.76
z10
6.12
6.04
MFT-1
z1
3.79
4.35
z2
3.77
4.79
z3
3.56
z4
3.73
3.95
z5
5.08
5.13
z6
3.45
3.33
z7
3.60
3.61
z8
4.42
4.12
z9
3.83
4.16
z10
4.33
3.85
z11
3.98
4.48
z12
4.02
3.51
LCT-B
z2
4.78
z8
5.32
5.30
4.59
z3
5.19
4.92
z4
5.36
4.65
z5
4.50
4.99
z6
4.66
z11
4.49
4.88
z7
4.46
4.62
z9
5.18
5.06
z10
4.64
4.19
SIMS δ18O spot
5.13 δ18O value
LA-ICPMS TEA spot
LA-ICPMS MI spot
25 100 (μm) 300

Figure S2

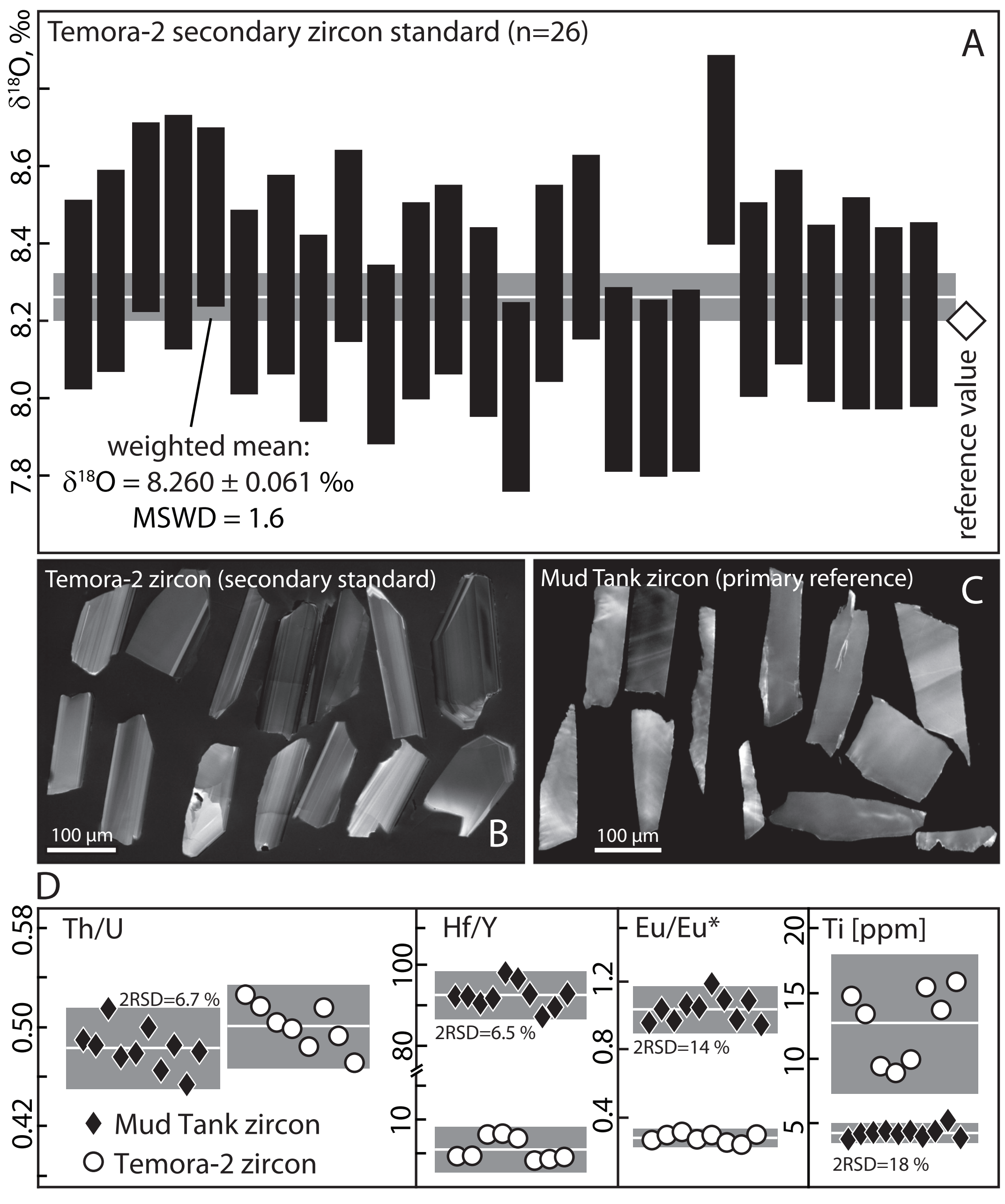

Temora-2 secondary zircon standard (n=26)
A
δ18O, ‰
8.6
8.4
8.2
8.0
7.8
weighted mean:
δ18O = 8.260 ± 0.061 ‰
MSWD = 1.6
reference value
Temora-2 zircon (secondary standard)
100 µm
B
Mud Tank zircon (primary reference)
C
100 µm
D
Th/U
0.58
0.50
0.42
2RSD=6.7 %
Mud Tank zircon
Temora-2 zircon
Hf/Y
100
80
10
2RSD=6.5 %
Eu/Eu*
1.2
0.8
0.4
2RSD=14 %
Ti [ppm]
20
15
10
5
2RSD=18 %

Figure S3

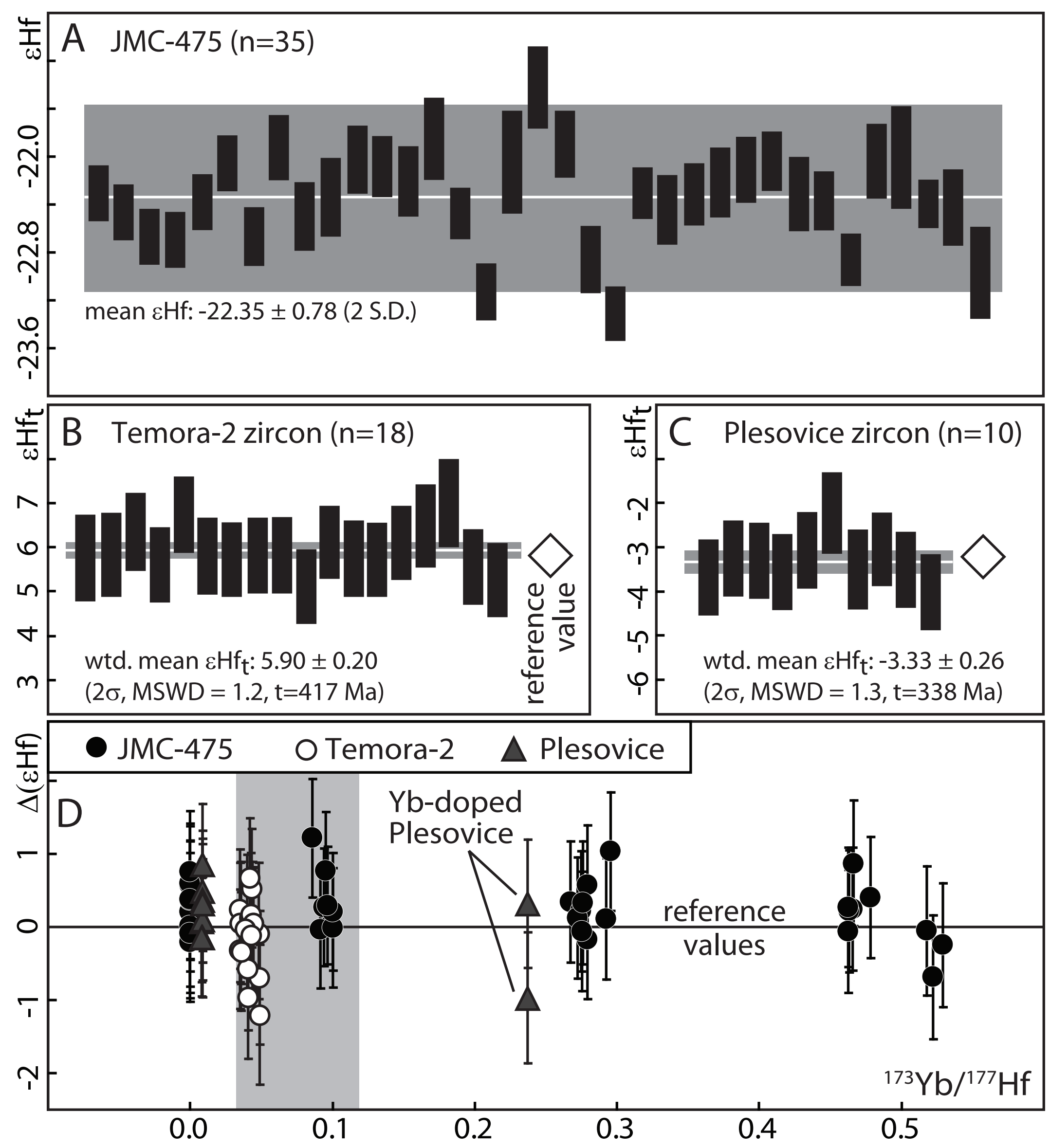

A JMC-475 (n=35)
εHf
-22.0
-22.8
-23.6
mean εHf: -22.35 ± 0.78 (2 S.D.)
B Temora-2 zircon (n=18)
εHf t
7
6
5
4
3
reference value
wtd. mean εHf t: 5.90 ± 0.20
(2σ, MSWD = 1.2, t=417 Ma)
C Plesovice zircon (n=10)
εHf t
-2
-3
-4
-5
-6
wtd. mean εHf t: -3.33 ± 0.26
(2σ, MSWD = 1.3, t=338 Ma)
JMC-475
Temora-2
Plesovice
Δ(εHf)
D
Yb-doped Plesovice
reference values
1
0
-1
-2
173Yb/177Hf
0.0
0.1
0.2
0.3
0.4
0.5

Figure S4

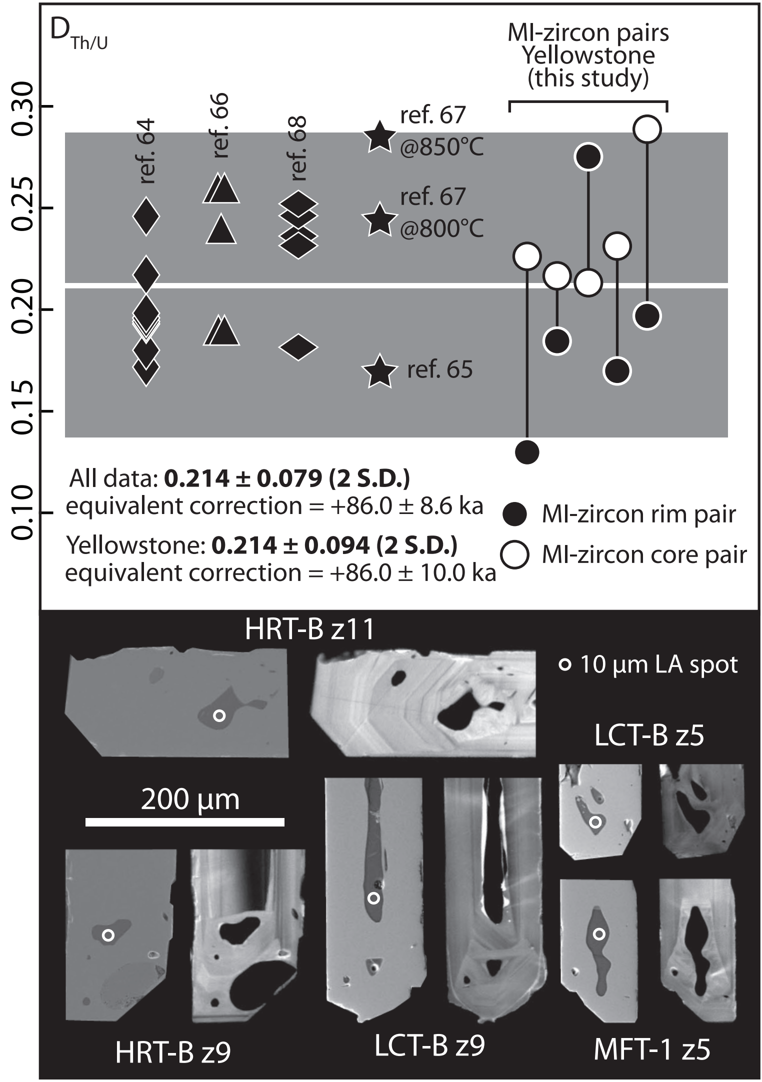

D Th/U
0.30
0.25
0.20
0.15
0.10
ref. 64
ref. 66
ref. 68
ref. 67 @850°C
ref. 67 @800°C
ref. 65
MI-zircon pairs Yellowstone (this study)
All data: **0.214 ± 0.079 (2 S.D.)**
equivalent correction = +86.0 ± 8.6 ka
Yellowstone: **0.214 ± 0.094 (2 S.D.)**
equivalent correction = +86.0 ± 10.0 ka
MI-zircon rim pair
MI-zircon core pair
HRT-B z11
10 μm LA spot
LCT-B z5
200 μm
HRT-B z9
LCT-B z9
MFT-1 z5

Figure S5

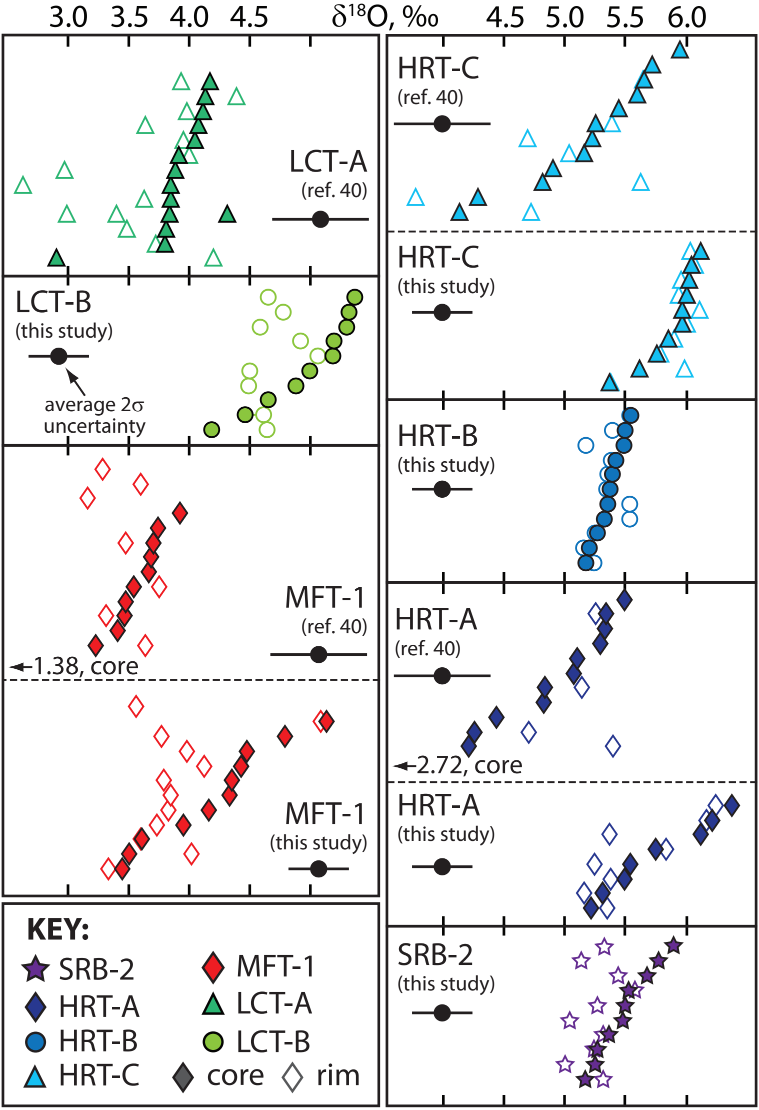

3.0 3.5 4.0 4.5
δ18O, ‰
4.5 5.0 5.5 6.0
LCT-A
(ref. 40)
LCT-B
(this study)
average 2σ uncertainty
MFT-1
(ref. 40)
←1.38, core
MFT-1
(this study)
HRT-C
(ref. 40)
HRT-C
(this study)
HRT-B
(this study)
HRT-A
(ref. 40)
←2.72, core
HRT-A
(this study)
SRB-2
(this study)
KEY:
SRB-2
HRT-A
HRT-B
HRT-C
MFT-1
LCT-A
LCT-B
core
rim
δ18O, ‰

Figure S6

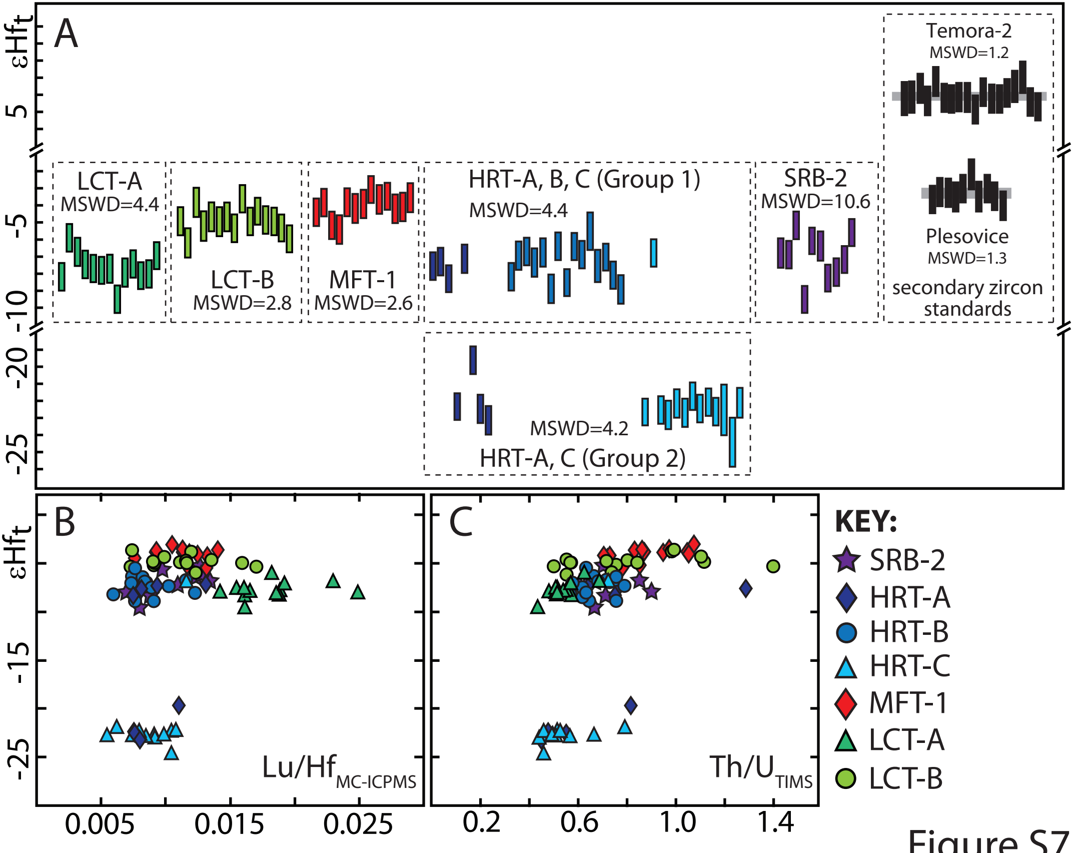
A
εHf_t
5
-5
-10
-20
-25
LCT-A
MSWD=4.4
LCT-B
MSWD=2.8
MFT-1
MSWD=2.6
HRT-A, B, C (Group 1)
MSWD=4.4
SRB-2
MSWD=10.6
Temora-2
MSWD=1.2
Plesovice
MSWD=1.3
secondary zircon standards
MSWD=4.2
HRT-A, C (Group 2)
B
C
εHf_t
-15
-25
Lu/Hf_MC-ICPMS
0.005
0.015
0.025
Th/U_TIMS
0.2
0.6
1.0
1.4
KEY:
SRB-2
HRT-A
HRT-B
HRT-C
MFT-1
LCT-A
LCT-B

Figure S7

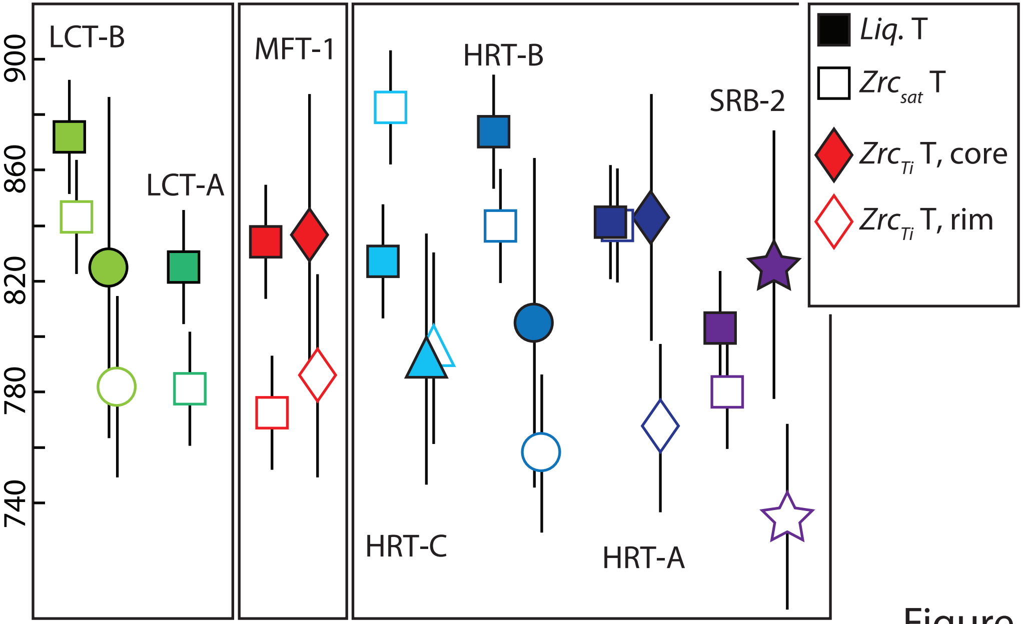
900
860
820
780
740
LCT-B
LCT-A
MFT-1
HRT-B
SRB-2
HRT-C
HRT-A
Liq. T
Zrc sat T
Zrc Ti T, core
Zrc Ti T, rim

Figure S8

Table S1

Sample	zrc	core/ rim	δ18O (‰)	2σ	MI Th/U	2σ	D (Th/U) MI-ZRC	Al2O3 wt%	P2O5 wt%	CaO wt%	Ti ppm	T [Ti-in-zircon] °C	Y ppm	Nb ppm	Ba ppm	La ppm	Ce ppm	Pr ppm	Nd ppm	Sm ppm	Eu ppm	Gd ppm	Tb ppm	Dy ppm	Ho ppm	Er ppm	Tm ppm	Yb ppm	Lu ppm	Hf ppm	Ta ppm	Th ppm	U ppm
LCT-B	z1.1	c	4.61	0.25				-	0.050	-	13.1	834	2280	5.74	-	0.24	9.57	0.601	9.13	15.8	3.41	72.3	21.9	269	80.6	386	69.4	507	99.3	7759	1.65	113	139
LCT-B	z1.2	r	3.45	0.24				-	0.056	-	8.65	790	947	12.7	-	0.45	17	0.149	1.62	2.52	0.136	17.3	5.96	80.9	28	136	31.1	246	46.2	9497	3.85	111	209
LCT-B	z2.1	c	5.32	0.24				0.002	0.086	-	29	930	1830	4.56	1.5	1.45	10.1	0.684	6.58	11.2	4.07	66	17.4	221	76.5	305	54.6	433	85.4	9582	1.31	81.3	103
LCT-B	z2.2	r	4.78	0.25				0.001	1.010	1.43	9.91	804	1350	11.6	2.23	216	551	70.1	329	64.5	2.34	91.5	17.3	162	48.8	211	39.4	330	63.3	9413	3.25	145	207
LCT-B	z3.1	c	5.19	0.24				-	0.051	-	12.9	833	2270	4.53	-	0.07	9.6	0.586	9.34	16.5	3.54	77.7	21.8	253	87.6	339	60.2	490	99.2	8734	1.26	91.5	105
LCT-B	z3.2	r	4.92	0.25				-	0.067	-	7.12	771	1090	11	-	0.03	19.6	0.135	2.74	5.47	0.433	29.8	8.47	111	39.2	181	33.3	288	58.1	9243	3.06	134	186
LCT-B	z4.1	c	5.36	0.23				-	0.057	-	18	871	2230	3.85	-	0.08	7.72	0.585	10.4	13.8	4.69	71.4	19.5	252	86.5	312	58.9	449	97.3	8353	0.913	83.9	94.2
LCT-B	z4.2	r	4.65	0.24				-	0.057	-	5.82	751	2010	7.6	-	0.02	16.5	0.356	5.88	12.3	1.16	61.2	17.6	229	70.7	344	64.3	514	81.1	10769	3	170	281
LCT-B	z5.1	c	4.99	0.23	3.14	0.50	0.21	-	0.031	-	10.1	806	1040	3.91	-	0.01	7.3	0.169	3.37	5.77	1	30.1	8.87	114	36.9	176	32.2	257	46	8819	1.29	41.8	62.3
LCT-B	z5.2	r	4.50	0.24			0.28	-	0.067	-	11.5	820	1470	16.4	-	0.02	21.7	0.217	3.21	6	0.838	37	10.9	145	52.2	260	44.7	382	70.2	10685	4.48	212	245
LCT-B	z6.2	c	4.66	0.24				-	0.054	-	10.1	806	2740	6.54	-	0.07	13.9	0.574	10.3	17.9	2.98	90.7	25.4	313	99.5	399	72.4	601	106	9073	2.09	141	166
LCT-B	z7.1	c	4.46	0.24				-	0.066	-	4.18	721	2710	12.6	-	0.03	22.5	0.189	3.25	9.97	0.338	60.5	21.2	275	95.1	465	90.4	730	130	12041	4.61	318	536
LCT-B	z7.2	r	4.62	0.25				-	0.058	-	4.9	735	1100	15.8	-	0.02	18.5	0.0945	1.76	4.02	0.23	24.2	7.85	108	39.7	189	38.9	319	60.1	11448	4.69	129	242
LCT-B	z8.1	c	5.30	0.24				-	0.045	-	15.7	855	2660	5.57	-	0.06	7.06	0.588	10.5	17.7	4.1	91.4	24.4	297	95.6	395	73.8	599	107	7717	1.48	99.1	122
LCT-B	z8.2	r	4.59	0.25				-	0.052	-	8.09	783	1020	9.69	-	0.05	14.9	0.164	2.23	4.04	0.568	23.9	6.87	89.3	36.1	146	31.4	274	47.5	8734	3.02	140	155
LCT-B	z9.1	c	5.18	0.28	4.69	0.35	0.23	-	0.070	-	14.8	848	4540	14.7	-	0.10	20.8	0.934	18.2	32.3	5.42	160	44.4	504	169	627	118	906	157	9158	3.16	236	217
LCT-B	z9.2	r	5.06	0.23			0.17	-	0.060	-	12.6	830	1050	7.4	-	0.01	11.6	0.158	2.82	6.07	0.849	32.4	9	109	37.9	173	32.5	279	52.1	8403	2.42	91	114
LCT-B	z10.1	c	4.19	0.25				-	0.076	-	5.25	741	2860	10	-	0.03	22.3	0.215	4.58	9.83	0.56	62.4	19.5	243	90.9	387	77.2	582	114	10854	3.77	308	425
LCT-B	z10.2	r	4.64	0.25				-	0.064	-	9.28	797	1140	10.9	-	0.03	19.1	0.2	2.79	5.84	0.461	29.5	8.95	108	42.1	173	33.5	287	57.9	8819	3.25	126	155
LCT-B	z11.1	c	4.88	0.23				1.220	0.064	0.086	168		1960	13.5	123	16	39.4	3.76	20.2	15.9	3.17	61.5	17.9	212	71.5	288	53.2	453	75.6	5648	2.06	114	124
LCT-B	z11.2	r	4.49	0.24				-	0.067	-	5.29	742	1140	18.3	-	0.01	18.1	0.0625	1.05	3.12	0.184	22.4	7.67	101	39.6	183	39.1	326	65.7	10515	5.19	147	279
MFT-1	z1.1	c	4.35	0.24				-	0.056	-	24.5	908	2170	3.2	-	0.08	5.37	0.704	9.73	17.4	4.94	71.3	20.2	238	75.5	305	60.3	505	92.5	7708	0.93	64.9	80.6
MFT-1	z1.2	r	3.79	0.25				-	0.053	-	5.64	748	876	10.7	-	-	14.9	0.0654	1.22	3.37	0.26	19.9	6.4	83.3	32.6	147	29.7	239	48	9413	3.48	127	193
MFT-1	z2.1	c	4.79	0.27				0.008	0.057	-	9.26	797	1810	7.17	-	0.74	17.2	0.438	5.52	9.21	1.2	48.6	14.4	170	72.2	289	50.9	424	78.3	8293	2.09	201	212
MFT-1	z2.2	r	3.77	0.26				-	0.083	0.030	6.57	763	1020	12.3	-	3.0	23.5	0.906	4.61	4.25	0.415	23.6	6.95	92.6	36.5	167	32.2	296	51.2	10515	3.64	134	199
MFT-1	z3.1	r	3.56	0.24				-	0.052	-	5.93	753	919	12.3	-	-	15.8	0.0633	1.29	3.04	0.221	19.2	6.81	90	34.7	152	31.1	263	52.2	10515	3.96	133	258
MFT-1	z4.1	c	3.95	0.25				-	0.050	-	20.9	889	2250	4.86	-	0.09	5.64	0.74	10.9	18	5.48	86.2	24.3	276	91.7	353	67.1	596	110	7920	1.09	97.7	103
MFT-1	z4.2	r	3.73	0.25				0.002	0.049	-	11.4	819	838	7.75	0.488	0.02	10.6	0.0757	1.41	3.16	0.589	19.5	7.17	87.7	28.5	134	27.2	242	48.1	9158	2.6	87.7	139
MFT-1	z5.1	c	5.13	0.23	3.33	0.37	0.29	-	0.046	-	20	883	2520	4.28	-	0.27	8.52	1.03	14.2	24.4	4.91	99.5	29.5	309	93.7	400	79.2	588	110	9243	1.3	105	109
MFT-1	z5.2	r	5.08	0.25			0.20	0.217	0.134	0.086	16.3	859	1100	13.9	6.17	20	65.3	6.37	29.3	11.4	0.556	31.8	9.01	110	40.4	173	34.7	283	55.5	9243	3.48	132	201
MFT-1	z6.1	c	3.45	0.24				-	0.043	-	12.8	832	2170	5.81	-	0.05	8.82	0.404	6.66	12.8	2.86	68.5	19.6	237	77.4	311	63.3	530	91.1	7912	1.67	104	140
MFT-1	z6.2	r	3.33	0.24				-	0.126	0.11	11.1	816	1050	11.7	-	17	55.8	4.93	25.2	7.48	0.423	25.2	7.93	97.4	38.5	164	31.7	302	60.6	10091	3.81	117	195
MFT-1	z7.1	c	3.61	0.25				-	0.311	0.40	8.18	784	2280	7.14	-	48	129	14.3	84.1	20.4	1.51	69.1	16.8	215	75.3	326	60.3	516	93.9	9497	2.57	149	207
MFT-1	z7.2	r	3.60	0.25				-	0.068	-	8.73	791	1010	13.5	-	0.93	19.6	0.362	2.96	4.02	0.405	24.5	7.67	97.1	37.7	172	33.9	313	57.7	11024	3.78	141	218
MFT-1	z8.1	c	4.42	0.25				-	0.052	-	15.1	850	3460	7.09	-	0.09	10.5	0.69	12.5	21.7	4.7	109	32.7	390	131	505	97.2	747	145	8166	2.09	176	218
MFT-1	z8.2	r	4.12	0.23				-	0.052	-	44.2	988	1400	8.82	0.413	0.17	10.5	0.243	4.23	7.36	1.22	37.9	10.6	141	51.3	201	41.3	370	67.6	7776	3.53	139	162
MFT-1	z9.1	c	4.16	0.26				0.018	0.130	0.063	12.2	827	3900	12.2	2.94	12	45.6	4.38	32.8	29.3	5.24	138	37.8	458	153	619	109	908	155	7445	2.95	267	270
MFT-1	z9.2	r	3.83	0.22				-	0.555	0.69	6.72	765	1030	10.8	-	130	313	36.5	181	37.7	1.22	51.9	10.7	112	36.6	159	31.6	277	52.3	10600	3.56	106	181
MFT-1	z10.1	c	4.33	0.24				-	0.107	-	15.1	850	4970	18.2	-	0.47	26.1	1.05	18.1	33	5.38	169	48.9	569	183	769	140	1200	204	8387	3.34	486	439
MFT-1	z10.2	r	3.85	0.24				-	0.104	-	5.54	746	1000	11.8	-	3.43	24.9	1.25	7.71	10.7	0.562	20.3	7.38	89.8	32.4	152	30	304	51.8	8904	3.35	135	215
MFT-1	z11.1	c	4.48	0.23				-	0.065	-	4.98	736	1740	6.08	-	0.45	15.3	0.364	5.8	8.82	0.804	43.2	14.3	176	62.8	265	51.8	450	78	8115	2	144	200
MFT-1	z11.2	r	3.98	0.23				-	0.074	-	10.2	807	1220	14.2	-	0.66	21.9	0.278	3.76	4.75	0.473	26	10.2	117	47.3	201	39.1	357	65.4	11024	4.3	169	256
MFT-1	z12.1	c	3.51	0.26				-	0.062	-	14.4	845	2620	6.05	-	0.08	9.56	0.663	10.2	17.9	3.29	85.6	24.4	260	95.3	394	70.6	617	113	7666	1.76	160	171
MFT-1	z12.2	r	4.02	0.27				0.004	0.053	-	7.67	778	1160	8.75	-	0.06	13.3	0.12	2.12	4.68	0.601	27.4	8.94	108	41.8	184	35.7	310	55.6	9413	2.8	94	138
HRT-A	z1.1	c	5.49	0.23				0.094	0.596	0.82	16.7	862	2250	6.92	2.82	89	233	29.9	142	41.4	2.76	92.2	23.2	267	80.7	332	63.3	514	94.7	8904	1.82	143	175
HRT-A	z1.2	r	5.38	0.24				-	0.046	-	7.83	780	973	9.41	-	0.01	15.1	0.0821	1.8	3.85	0.398	23.2	7.83	96.6	34.7	154	31.4	270	51.8	11109	2.84	101	177
HRT-A	z2.1	c	5.54	0.24				-	0.061	-	19.2	878	2170	6.29	-	0.72	12	0.528	6.82	12.8	3.01	66.5	19.7	246	79.4	339	62.7	518	96.5	9073	1.96	124	163
HRT-A	z2.2	r	5.24	0.24				-	0.056	-	7.36	774	1070	11.2	-	0.01	17.1	0.0905	1.92	4.64	0.396	25.9	8.72	111	37.4	170	33.4	298	57.7	11024	3.44	121	226
HRT-A	z3.1	c	6.12	0.23				-	0.050	-	18	871	1490	1.85	-	0.12	3.5	0.466	5.86	10.6	3.86	46.3	14.1	162	50.2	216	38.5	337	64.6	6487	0.554	72	125
HRT-A	z3.2	r	5.36	0.38				-	0.038	-	46.8		507	2.97	-	0.03	3.99	0.0472	0.843	1.99	0.795	12.8	4.38	53.6	18.4	79.3	15.7	144	28.1	7377	0.754	33.7	94
HRT-A	z4.1	c	5.31	0.25				-	0.060	-	5.86	752	1890	6.36	-	0.53	15.1	0.336	4.75	8.2	1.29	51.9	15.9	197	69.4	300	56.4	469	87.9	10091	2.2	147	221
HRT-A	z4.2	r	5.15	0.23				-	0.060	-	5.82	751	2500	10	-	0.06	19.2	0.203	4.16	10	0.711	56.3	18.5	243	85.4	372	75.6	601	111	12211	3.6	234	407
HRT-A	z5.1	c	5.75	0.25				-	0.222	-	13.4	837	8300	48.4	0.303	0.16	118	1.13	22.7	44	3.1	248	75.4	984	300	1150	212	1870	342	10939	11.7	1930	2410
HRT-A	z5.2	r	5.84	0.26				0.002	0.038	-	83.6		806	6.04	-	0.05	11.9	0.061	1.05	3.13	0.505	19	6.13	81.5	28.8	124	23.6	220	40.9	9667	2	83.9	248
HRT-A	z6.1	c	5.21	0.25				-	0.061	-	15.8	856	3130	7.13	-	1.14	13.2	0.894	10.8	18.6	4.04	104	30.5	356	117	480	84.8	749	136	9752	2.17	170	227
HRT-A	z6.2	r	5.35	0.27				-	0.059	-	4.45	726	1390	25.4	0.182	0.08	20.3	0.12	1.72	4.07	0.274	25.8	9.23	127	46.8	222	43.9	384	70.2	11448	6.86	194	426
HRT-A	z7.1	c	6.37	0.30				0.051	0.053	-	19.3	879	1200	1.82	6.02	1.59	4.91	0.5	5.27	7.53	2.75	37.7	11.4	123	41.9	168	31.5	280	52.6	6309	0.596	50.5	100
HRT-A	z7.2	r	6.24	0.25				0.125	0.156	0.18	384		652	14.1	1.32	9.42	26.3	3.05	15.2	5.96	1.5	19	5.76	67.9	22.9	103	19.6	181	34.7	8208	1.19	45.8	121
HRT-A	z9.1	c	6.21	0.25				-	0.042	-	10.3	808	1230	1.93	-	0.07	4.24	0.325	4.54	7.38	2.37	37.4	10.7	125	42.9	177	32.8	260	51.2	6953	0.676	57.7	119
HRT-A	z9.2	r	6.17	0.24				-	0.042	-	9.99	805	629	3	-	0.01	5.34	0.0504	0.955	2.79	0.844	15.7	5.49	62	21.3	96	18.7	170	34	7522	0.884	46.6	120
HRT-B	z1.1	c	5.20	0.23				-	0.071	-	4.94	736	2060	10.6	-	0.24	24.3	0.137	2.54	6.8	0.44	43.7	14.5	189	68	309	63	591	101	11872	3.84	283	434
HRT-B	z1.2	r	5.16	0.23				0.013	0.061	-	6.57	763	1230	17.1	0.422	0.33	18.5	0.137	1.59	3.41	0.204	23.3	8.11	110	41.6	197	38.3	333	61.6	12465	5.62	176	392
HRT-B	z2.1	c	5.50	0.23				-	0.049	-	13.7	839	1920	3.07	-	0.04	6.7	0.417	6.96	12.9	2.73	63.5	18.1	208	69.6	291	52.9	444	83.4	8819	1.05	80.5	110
HRT-B	z2.2	r	5.40	0.22				-	0.064	-	10	805	949	4.88	0.23	0.04	6.69	0.12	1.97	4.52	0.754	24.3	7.31	95.4	33.9	151	27.4	244	45.9	7912	1.85	51.8	89.8
HRT-B	z3.1	c	5.37	0.27				-	0.043	-	17.8	869	1480	3.17	0.0332	0.06	5.6	0.377	6.08	10.1	3.97	48.5	15	164	53.3	224	44.2	367	67.2	7818	0.948	58.5	83.3
HRT-B	z3.2	r	5.35	0.24				-	0.048	-	5.71	749	957	12	-	0.01	16.2	0.0603	1.27	2.95	0.33	21.3	7.24	89.7	33.5	153	31.6	274	50.6	10600	3.82	114	208
HRT-B	z4.1	c	5.27	0.22				-	0.076	-	21	889	3150	4.9	-	0.09	7.6	0.835	14.8	25.1	8.36	115	33.6	363	116	449	87.5	726	133	6741	1.12	147	150
HRT-B	z4.2	r	5.25	0.23				-	0.060	-	5.34	743	1310	15.3	-	0.06	21.2	0.148	1.77	3.98	0.431	29	9.1	126	44.5	203	40.4	350	63.3	11617	4.49	168	305
HRT-B	z5.1	c	5.49	0.25				-	0.083	-	10.1	806	3610	9.47	-	0.07	17.8	0.707	12.7	23.3	4.64	114	34.5	384	129	531	103	859	153	7717	2.19	340	318
HRT-B	z5.2	r	5.17	0.23				0.004	0.075	-	3.53	706	727	9.12	-	0.41	14.6	0.211	1.4	2.41	0.17	13.8	5.16	64.9	25.1	120	24.2	223	40.3	9073	2.99	105	211
HRT-B	z6.1	c	5.33	0.23				-	0.059	-	9.26	797	2580	7.04	-	0.05	13.5	0.477	7.93	15.5	2.46	80.3	24.9	280	91.9	379	75.2	629	108	9243	2.23	182	230
HRT-B	z6.2	r	5.53	0.23				-	0.065	-	7	769	1100	12.6	-	0.01	17.3	0.101	2	4.23	0.387	25.1	8.32	107	38.2	172	36.1	305	57	10345	3.72	127	231
HRT-B	z7.1	c	5.39	0.25				-	0.049	-	10.7	812	1240	2.2	0.159	0.03	3.79	0.267	4.63	7.81	2.17	39.8	12.1	133	44	193	34.9	293	53.2	6580	0.711	45.8	62.1
HRT-B	z7.2	r	5.36	0.24				-	0.047	-	5.14	739	767	10.7	-	-	14	0.046	1.02	2.41	0.226	16.8	5.84	76.3	27.3	124	26.7	222	42.8	10685	3.3	82.6	169
HRT-B	z8.1	c	5.35	0.27				-	0.055	-	7.65	778	2250	5.2	-	0.047	9.64	0.367	5.97	13.8	2.88	69.8	20.6	244	77.1	327	61.6	500	95.1	7437	1.57	122	152
HRT-B	z8.2	r	5.53	0.23				-	0.060	-	8.21	785	912	8.8	-	0.016	15.3	0.0685	1.51	3.62	0.444	21.8	7.15	89.2	32.3	143	28.2	254	48.5	9837	2.74	97.9	169
HRT-B	z9.1	c	5.54	0.24	3.73	0.13	0.23	0.497	0.669	0.74	26.4		1990	6.53	20.5	51.9	128	17.1	83.6	27.3	3.33	74.5	20.1	219	73.7	296	56.1	459	87.2	6869	1.87	104	123
HRT-B	z9.2	r	5.54	0.22			0.13	-	0.059	-	5.65	748	973	11.2	-	-	15.4	0.0465	1.07	3.17	0.24	18.5	6.85	86.1	31.3	149	30.7	273	53	11533	3.69	102	210
HRT-B	z10.1	c	5.43	0.24				-	0.048	-	13.1	834	2050	4.12	-	0.04	7.05	0.413	6.92	12.9	3.01	64	19	222	71	307	57	457	84.7	8649	1.32	80.2	120
HRT-B	z10.2	r	5.39	0.22				-	0.060	-	8.6	790	886	6.07	-	0.01	10.5	0.0934	1.79	4.27	0.558	21.2	6.71	85.1	29.5	131	27.2	234	44.1	7996	1.96	76.6	127
HRT-B	z11.1	c	5.18	0.24	2.58	0.17	0.22	-	0.066	-	3	692	1400	17.2	-	0.03	22	0.0891	1.92	4.09	0.325	27.5	9.54	124	48	223	46.4	396	74.9	11193	5.24	232	414
HRT-B	z11.2	r	5.24	0.23			0.19	-	0.052	-	5.05	738	1070	15.6	-	0.04	17.1	0.0683	1.52	3.57	0.236	21.9	7.86	99.3	39.3	176	35	309	60.4	11278	4.89	150	314
HRT-C	z1.1	c	6.05	0.23				-	0.061	0.032	17.4	867	1630	2.43	-	1.3	7.18	0.878	7.91	13.3	4.13	60.6	16	177	62	250	47.8	400	74.7	6623	0.681	77.1	159
HRT-C	z1.2	r	6.07	0.23				-	0.047	-	10.4	809	623	3.09	-	-	4.58	0.0846	1.21	2.17	0.798	15.4	4.96	62.4	21.2	103	20.9	154	29.7	8005	0.833	37.4	104
HRT-C	z2.1	c	5.36	0.23				-	0.061	-	10.5	810	2600	5.41	-	0.044	8.9	0.511	8.02	15.8	3.69	82.9	24.8	299	99.9	408	75.3	567	104	8819	1.76	127	174
HRT-C	z2.2	r	5.37	0.25				-	0.063	-	9.37	798	1660	9.66	-	0.018	13	0.216	3.64	6.35	1.42	40.3	13.5	155	59.8	247	45.4	383	70.1	9413	3	148	212
HRT-C	z3.1	c	6.02	0.26				-	0.042	-	9.92	804	1640	2.48	-	0.018	7.08	0.227	4.44	10.2	2.51	54	14.4	173	62.6	260	44.7	382	67	9243	0.751	91.5	168
HRT-C	z3.2	r	5.95	0.23				-	0.047	-	9.56	800	787	3.95	0.142	-	8.35	0.0781	1.3	3.08	0.815	20.2	5.41	72.2	28.2	123	24.4	215	37.8	9073	1.4	59.5	161
HRT-C	z4.1	c	5.96	0.24				-	0.075	-	5.9	752	2510	9.11	-	0.028	18.7	0.325	6.14	11.7	1.79	67	21	257	93	405	77.2	632	106	9921	2.75	288	645
HRT-C	z4.2	r	6.00	0.23				-	0.049	-	6.11	756	990	6.52	-	-	11.4	0.0657	1.41	3.41	0.604	23.6	8.03	89.8	34.1	148	29.2	260	46.9	9752	2.07	110	270
HRT-C	z5.1	c	5.85	0.25				-	0.063	-	4.38	725	1760	4.01	-	0.026	10.7	0.362	6.24	10.6	1.73	55.4	15.8	183	64.1	271	49.1	417	75.2	8819	1.31	126	266
HRT-C	z5.2	r	5.90	0.24				-	0.050	-	6.94	768	859	4.62	-	-	8.61	0.0508	1.19	3.65	0.625	23.7	6.97	88.5	32	144	26.9	238	45.3	8454	1.56	68.8	171
HRT-C	z6.1	c	5.96	0.22				-	0.034	-	12.7	831	1330	2.35	-	0.012	5.15	0.206	4.34	8.28	2.84	46.6	13.6	146	49.8	209	38.5	308	57.3	8649	0.766	64	142
HRT-C	z6.2	r	6.10	0.24				-	0.040	-	14.6	847	1650	2.35	-	0.029	6.06	0.308	6.15	11	3.51	57.8	16.5	165	57	246	46.2	387	67.7	8565	0.83	78.9	159
HRT-C	z7.1	c	5.62	0.22				0.268	0.122	0.12	670		1080	5.55	40.9	8	40.9	2.82	14.3	7.82	1.81	28	8.39	102	37.6	175	36.1	310	63.3	8115	1.29	278	316
HRT-C	z7.2	r	5.99	0.25				-	0.028	-	8.9	793	647	3.64	-	-	5.75	0.0447	1.08	2.46	0.704	16.2	5.05	57.2	22.8	102	18.2	168	31.8	8327	1.01	45.2	126
HRT-C	z8.1	c	6.00	0.22				-	0.037	-	8.8	792	1560	2.62	-	0.024	6.48	0.34	6.09	12.4	2.88	53.6	15.1	164	58.4	237	40.2	369	67.9	8073	0.878	86.8	159
HRT-C	z8.2	r	5.94	0.23				-	0.027	-	12	825	633	3.3	-	-	6.31	0.0504	1.04	2.57	0.72	14.2	5.2	61.8	22.3	98.8	18.9	176	30.8	9328	0.971	44.2	124
HRT-C	z9.1	c	5.76	0.25				-	0.056	-	5.69	749	1830	8.53	-	0.022	14.4	0.227	4.15	8.35	1.28	46.3	16.4	177	62	273	53.1	503	82.5	9837	2.62	201	444
HRT-C	z9.2	r	5.79	0.23				-	0.046	-	4.68	731	1140	8.12	-	-	14.6	0.0863	1.77	4.39	0.621	26.7	9.13	110	39.5	169	32.7	303	54.8	10430	2.51	135	347
HRT-C	z10.1	c	6.12	0.24				-	0.054	-	7.17	771	2220	6.57	-	0.073	16.5	0.272	5.54	11.3	1.47	64	20	234	78.5	326	58.5	517	91	9582	2.08	214	419
HRT-C	z10.3	r	6.04	0.25				-	0.028	-	8.29	786	684	4.45	-	0.24	8.59	0.116	1.21	2.73	0.672	16.4	5.11	68.7	24.7	108	20.5	179	34.3	9413	1.12	48.4	146
SRB-2	z1.1	c	5.36	0.23				-	0.075	-	11.2	817	5260	16.1	-	0.097	22.2	1.09	20.4	37.3	7.49	187	52.5	627	193	753	140	1130	191	8022	2.66	369	339
SRB-2	z1.2	r	5.31	0.24				0.003	0.059	-	9.32	798	3780	10.8	0.56	0.42	13.3	0.796	11.9	22.7	4.76	122	36.2	417	135	555	98.2	858	149	7827	2.44	224	273
SRB-2	z2.1	c	5.53	0.25				-	0.063	-	19.4	880	2540	4.1	-	0.98	7.74	0.932	12.9	19.3	5.87	85.9	26.3	294	89.5	387	78.8	648	117	7912	1.22	124	162
SRB-2	z2.2	r	5.57	0.23				-	0.056	-	2.38	673	796	10.6	0.777	0.074	12.9	0.0465	1.19	2.22	0.286	15.7	5.49	67.8	28.1	128	27.6	239	47.7	9413	3.58	131	244
SRB-2	z3.1	c	5.50	0.23				-	0.056	-	18.7	875	3570	6.58	-	0.089	8.37	0.76	14.7	25.4	7.09	130	34.9	411	145	594	111	943	168	8446	1.94	189	212
SRB-2	z3.2	r	5.27	0.25				0.003	0.038	-	3.63	708	607	9.77	0.249	0.018	11	0.0249	0.38	1.39	0.0992	10.6	3.74	54.4	18.9	98.8	20.4	194	37.5	12211	3.59	71	187
SRB-2	z4.1	c	5.90	0.24				-	0.059	-	14	842	1940	3.18	-	0.048	6.81	0.399	6.53	11.5	2.63	60.6	18	210	68.7	295	56.8	480	84.7	8251	1.15	110	165
SRB-2	z4.2	r	5.32	0.24				0.008	0.154	0.097	5.01	737	1260	15.9	-	18	64.8	6.13	29.3	9.91	0.378	28.8	9.32	125	43.1	199	41.7	383	68.5	11024	4.82	240	470
SRB-2	z5.1	c	5.48	0.25				-	0.089	-	9.88	804	4750	15.5	-	0.18	21.9	1.05	20	33.8	7.98	175	51.6	589	188	742	133	1120	197	9243	2.98	384	331
SRB-2	z5.2	r	5.05	0.23				-	0.066	-	5.68	749	1160	17.3	0.321	0.08	16.5	0.0512	1.27	3.15	0.244	21.7	7.94	104	39.9	187	36.2	350	63.3	10006	5.32	227	441
SRB-2	z6.1	c	5.68	0.26				-	0.046	-	15.3	852	1490	2.67	-	0.03	5.77	0.3	4.65	9.22	2.09	43.5	14	156	53.7	219	43.7	384	70.7	8310	0.913	86.1	129
SRB-2	z6.2	r	5.44	0.22				-	0.063	-	5.65	748	1210	13.1	-	0.01	17.4	0.0866	1.47	3.84	0.487	24.7	8.53	116	42	197	38	337	67	10345	4.13	174	322
SRB-2	z7.1	c	5.78	0.23				-	0.055	-	19.8	882	2380	3.44	-	0.07	6.52	0.468	7.52	13.1	3.1	68.2	20.9	254	82.1	358	65.2	557	101	8565	1.24	138	195
SRB-2	z7.2	r	5.14	0.25				0.041	1.410	2.1	6.4	760	1320	11.5	1.8	443	1120	148	653	140	3.94	149	23.2	180	46.7	188	35	299	55.2	11024	4.03	180	321
SRB-2	z8.1	c	5.18	0.24				-	0.066	-	5.91	753	1390	11.4	-	0.010	20.5	0.11	2.14	4.7	0.431	28.1	9.75	124	47.7	225	44.2	399	73.9	10345	3.81	267	368
SRB-2	z8.2	r	5.32	0.23				-	0.050	-	5.04	738	929	10.3	-	-	14.6	0.0612	1.22	2.99	0.356	18.4	6.97	88.8	32.5	154	29.8	279	52.7	10685	3.37	123	219
SRB-2	z9.2	r	5.01	0.23				0.002	0.064	-	4.11	719	1260	15.9	-	0.04	19.3	0.0638	1.37	3.27	0.257	24.1	8.53	114	42.8	204	41.4	386	68.7	11787	5.05	250	468
SRB-2	z9.3	c	5.25	0.24				0.004	0.064	-	9.51	800	2060	5.51	0.641	1.8	13.8	0.94	9.12	11.5	2.44	56.8	18.1	213	70.6	313	59.7	534	97	7784	1.65	159	251
SRB-2	z10.1	c	5.27	0.23				-	0.061	-	6.4	760	1320	10.2	-	0.06	20	0.111	1.93	4.09	0.433	27.6	9.37	125	47.4	220	44.4	411	74.8	12041	3.54	239	386
SRB-2	z10.2	r	5.24	0.24				0.005	0.195	0.21	4.37	725	1240	15.1	0.465	25	83.7	7.99	38.6	12	0.615	31.4	9.41	121	44.3	202	38.4	355	68.5	11787	4.87	182	333
MudTank	1.1						1	-	0.014	-	3.78	691	105	2.39	-	0.0066	1.07	0.072	0.94	1.41	0.83	5.43	1.44	14.1	3.84	14.0	2.67	22.3	3.29	9667	2.95	14	28.6
MudTank	1.2						2	-	0.012	-	4.21	700	105	2.27	-	0.0072	1.03	0.067	0.90	1.36	0.91	5.67	1.54	13.6	3.90	14.3	2.55	20.1	3.41	9667	2.99	14.3	29.5
MudTank	1.3						3	-	0.012	-	4.36	703	105	2.47	-	0.0066	1.07	0.066	0.91	1.51	0.89	5.64	1.44	14.5	4.25	15.0	2.63	22.3	3.43	9497	3.12	15.4	29.9
MudTank	1.4						4	-	0.012	-	4.46	705	102	2.26	-	-	1.04	0.060	0.92	1.38	0.91	5.33	1.36	13.0	3.98	14.4	2.62	19.6	3.38	9413	3.06	13.8	29
MudTank	1.5						5	-	0.010	-	4.2	700	98.6	2.16	-	0.0125	1.01	0.062	0.86	1.32	0.88	5.35	1.39	14.0	3.97	14.3	2.42	19.3	3.26	9667	2.82	13.7	28.6
MudTank	1.6						6	-	0.011	-	4.39	704	98.4	2.12	-	0.0122	1.07	0.055	0.92	1.37	0.98	4.96	1.27	13.8	3.84	14.8	2.31	18.7	3.19	9497	3.01	14.2	28.4
MudTank	1.7						7	-	0.014	-	4.05	697	107	2.38	-	-	1.09	0.068	0.90	1.30	0.91	5.30	1.50	14.9	4.27	15.4	2.62	21.2	3.48	9921	3.15	14.6	31.4
MudTank	1.8						8	-	0.013	-	4.39	704	110	2.48	-	0.0109	1.11	0.068	1.14	1.44	0.84	5.18	1.54	15.1	4.49	15.1	2.77	21.0	3.54	9582	3.07	15	30.9
MudTank	1.9						9	-	0.016	-	5.28	720	107	2.33	-	-	0.98	0.058	0.70	1.17	0.88	5.68	1.44	13.6	3.89	14.0	2.24	18.7	3.4	9582	2.73	13.6	30
MudTank	1.10						10	-	0.009	-	3.91	694	105	2.37	-	-	1.08	0.070	0.97	1.51	0.84	5.23	1.47	13.0	3.96	15.4	2.49	19.0	3.1	9752	3.15	13.5	28.1
Average							11				4.30	701.73	104.30	2.32		0.01	1.06	0.06	0.92	1.38	0.89	5.4	1.4	14.0	4.0	14.7	2.5	20	3.3	9625	3.0	14	29
2 SD (abs)											0.78	14.97	6.97	0.23		0.01	0.07	0.01	0.20	0.19	0.08	0.44	0.16	1.36	0.42	1.02	0.32	2.65	0.26	276.08	0.27	1.19	2.10
2 SD (%)											18.1	2.1	6.7	9.8		55.3	7.0	16.9	22.3	14.0	9.1	8.3	11.0	9.7	10.3	7.0	12.5	13.1	7.7	2.9	8.8	8.4	7.1
Temora	1.1						12	-	0.040	-	14.7	821	1630	0.937	-	0.0202	3.17	0.28	3.54	6.54	1.32	36.8	12.1	155	53.6	255	48.5	446	85.1	7208	0.259	62	118
Temora	1.2						13	-	0.042	-	13.4	811	1600	0.842	-	0.0142	3.07	0.226	3.8	6.09	1.45	39.1	11.9	150	54.1	253	46.3	427	75	7386	0.23	58.9	114
Temora	2.1						14	-	0.035	-	9.35	774	1090	0.771	-	-	3.47	0.143	2.54	4.59	1.03	23.2	7.34	97.2	35	172	33.3	324	61	8378	0.197	90.1	179
Temora	2.2						15	-	0.025	-	8.82	768	1000	0.711	-	-	3.09	0.116	2.22	3.91	0.775	20.8	6.76	83.7	30.9	147	28.9	286	56.4	7886	0.114	81.6	164
Temora	2.3						16	-	0.028	-	9.85	779	1230	0.849	-	-	3.56	0.163	2.83	4.92	1.03	24.3	7.71	111	36.8	170	35	345	64.9	8904	0.156	93.4	193
Temora	3.1						17	-	0.046	-	15.4	826	1840	0.956	-	0.0305	3.47	0.283	4.07	5.97	1.12	33.3	12.7	159	57.4	285	54.6	508	96	7174	0.349	61.9	120
Temora	3.2						18	-	0.044	-	13.7	813	1690	0.953	-	0.0258	3.38	0.257	4.13	6.14	1.07	32.9	11.6	149	57.1	269	52.1	500	92.1	6970	0.291	56.6	115
Temora	3.3						19	-	0.045	-	15.8	828	1700	0.99	-	0.0305	3.48	0.239	4.13	5.58	1.29	32	10.6	140	55.4	250	49.5	485	90.3	7471	0.31	56.5	120
Average							20				12.63	802.33	1472.5	0.88		0.02	3.34	0.21	3.41	5.47	1.14	30	10	131	48	225	44	415	78	7672	0	70	140
2 SD (abs)											5.33	46.32	593.11	0.19		0.01	0.37	0.12	1.44	1.70	0.40	12.55	4.53	54.33	20.97	99.41	18.10	160.78	28.80	1253	0.15	29.16	61.20
2 SD (%)											42.2	5.8	40.3	21.2		51.9	11.0	56.4	42.3	31.0	34.9	41.4	44.9	41.6	44.1	44.2	41.6	38.7	37.1	16.3	62.7	41.6	43.6

Table S2

	Dates (Ma)							Composition				Isotopic Ratios							Lutetuim-hafnium isotopes							
Sample	206Pb/ 238U (a)	±2σ abs	206Pb/ 238U <Th> (b)	±2σ abs	207Pb/ 235U (a)	±2σ abs	Corr. coef.	Th/ U (d)	Pb* (pg) (e)	Pbc (pg) (f)	Pb*/ Pbc (g)	206Pb/ 204Pb (i)	206Pb/ 238U (j)	±2σ %	207Pb/ 235U (j)	±2σ %	207Pb/ 206Pb (j)	±2σ %	Yb/ Hf	Lu/ Hf	176Hf/ 177Hf (0)	±2σ	176Hf/ 177Hf (t)	±2σ	εHf	±2σ
LCT-A z1	0.5389	0.0072	0.625	0.011	0.86	0.18	0.757	0.57	0.32	0.30	1.08	76	0.0000836	1.33	0.0008476	21.4	0.0735589	20.4	0.116	0.016	0.282552	0.000006	0.282552	0.000006	-8.22	0.81
LCT-A z2	0.5419	0.0014	0.6280	0.0088	0.73	0.04	0.752	0.63	0.54	0.09	5.84	330	0.0000841	0.26	0.0007237	4.8	0.0624600	4.6	0.141	0.018	0.282617	0.000005	0.282617	0.000005	-5.94	0.80
LCT-A z3	0.5386	0.0022	0.6246	0.0089	0.70	0.04	0.777	0.69	0.37	0.09	3.97	217	0.0000836	0.41	0.0006890	5.3	0.0598324	5.0	0.153	0.023	0.282593	0.000007	0.282593	0.000007	-6.77	0.82
LCT-A z5	0.5388	0.0017	0.6248	0.0088	0.72	0.04	0.755	0.51	0.61	0.11	5.63	329	0.0000836	0.31	0.0007063	5.2	0.0613171	4.9	0.120	0.016	0.282572	0.000004	0.282572	0.000004	-7.51	0.79
LCT-A z7	0.5402	0.0023	0.6262	0.0089	0.76	0.05	0.744	0.55	0.44	0.11	3.86	228	0.0000838	0.42	0.0007486	7.0	0.0648184	6.7	0.136	0.019	0.282566	0.000004	0.282566	0.000004	-7.73	0.80
LCT-A z8	0.5484	0.0042	0.6344	0.0096	0.82	0.11	0.754	0.48	0.43	0.23	1.86	121	0.0000851	0.76	0.0008047	13.0	0.0686419	12.5	0.105	0.014	0.282563	0.000005	0.282563	0.000005	-7.84	0.80
LCT-A z9	0.5444	0.0044	0.6305	0.0097	0.78	0.11	0.774	0.54	0.41	0.21	1.88	121	0.0000845	0.81	0.0007660	14.1	0.0658105	13.5	0.123	0.017	0.282565	0.000005	0.282565	0.000005	-7.77	0.80
LCT-A z10	0.5365	0.0034	0.6225	0.0093	0.67	0.08	0.767	0.44	0.23	0.09	2.56	163	0.0000832	0.64	0.0006602	12.0	0.0575580	11.5	0.107	0.016	0.282515	0.000005	0.282515	0.000005	-9.52	0.80
LCT-A z11	0.5334	0.0035	0.6195	0.0093	0.66	0.09	0.759	0.51	0.25	0.09	2.67	155	0.0000828	0.65	0.0006489	13.4	0.0568959	12.9	0.164	0.025	0.282559	0.000008	0.282559	0.000008	-7.98	0.83
LCT-A z12	0.5433	0.0019	0.6294	0.0088	0.74	0.05	0.728	0.51	0.56	0.13	4.41	262	0.0000843	0.34	0.0007275	6.1	0.0626288	5.9	0.126	0.016	0.282573	0.000004	0.282573	0.000004	-7.48	0.79
LCT-A z13	0.5412	0.0026	0.6272	0.0090	0.71	0.06	0.756	0.51	0.32	0.09	3.38	205	0.0000839	0.48	0.0006972	8.5	0.0602619	8.2	0.126	0.019	0.282554	0.000005	0.282554	0.000005	-8.16	0.80
LCT-A z14	0.5349	0.0043	0.6209	0.0097	0.73	0.10	0.764	0.51	0.27	0.14	1.96	126	0.0000830	0.80	0.0007220	14.0	0.0631396	13.4	0.124	0.019	0.282557	0.000005	0.282557	0.000005	-8.05	0.80
LCT-A z15	0.5393	0.0012	0.6253	0.0087	0.72	0.03	0.742	0.57	0.92	0.13	7.16	407	0.0000837	0.23	0.0007077	3.9	0.0613792	3.8	0.150	0.019	0.282587	0.000003	0.282587	0.000003	-6.99	0.79
LCT-B z2	0.535	0.031	0.621	0.032	1.09	0.82	0.754	1.12	0.06	0.20	0.28	31	0.0000831	5.82	0.0010768	74.8	0.0940697	70.5	0.069	0.011	0.282645	0.000008	0.282645	0.000008	-4.95	0.83
LCT-B z3	0.5430	0.0035	0.6291	0.0093	0.78	0.09	0.750	0.55	0.79	0.35	2.21	138	0.0000842	0.65	0.0007699	11.5	0.0663138	11.0	0.047	0.008	0.282608	0.000009	0.282608	0.000009	-6.26	0.84
LCT-B z4	0.522	0.030	0.608	0.031	0.95	0.79	0.755	0.98	0.06	0.21	0.27	31	0.0000810	5.76	0.0009329	83.7	0.0835689	79.5	0.075	0.012	0.282676	0.000010	0.282676	0.000010	-3.85	0.85
LCT-B z5	0.519	0.024	0.605	0.025	0.72	0.62	0.765	0.76	0.07	0.20	0.33	36	0.0000805	4.60	0.0007103	86.2	0.0639931	82.7	0.057	0.009	0.282636	0.000011	0.282636	0.000011	-5.25	0.87
LCT-B z6	0.5524	0.0089	0.638	0.012	0.54	0.22	0.783	0.80	0.10	0.10	1.05	75	0.0000857	1.61	0.0005299	40.3	0.0448677	39.1	0.081	0.012	0.282650	0.000008	0.282650	0.000008	-4.74	0.83
LCT-B z7	0.549	0.019	0.635	0.021	0.75	0.50	0.751	0.57	0.23	0.58	0.39	41	0.0000852	3.40	0.0007404	66.6	0.0630265	64.1	0.064	0.009	0.282643	0.000008	0.282643	0.000008	-5.02	0.83
LCT-B z8	0.5420	0.0037	0.6281	0.0094	0.81	0.09	0.755	0.56	0.38	0.18	2.13	133	0.0000841	0.68	0.0008013	11.6	0.0691452	11.1	0.085	0.014	0.282651	0.000009	0.282651	0.000009	-4.74	0.84
LCT-B z9	0.547	0.024	0.633	0.026	1.24	0.48	0.794	1.40	0.07	0.19	0.37	32	0.0000849	4.44	0.0012181	38.8	0.1041210	35.4	0.111	0.017	0.282632	0.000007	0.282632	0.000007	-5.40	0.82
LCT-B z10	0.552	0.028	0.638	0.030	1.19	0.75	0.752	0.99	0.28	0.92	0.31	32	0.0000856	5.12	0.0011740	62.8	0.0994716	59.0	0.058	0.007	0.282681	0.000003	0.282681	0.000003	-3.67	0.79
LCT-B z11	0.568	0.013	0.654	0.016	1.00	0.34	0.754	0.84	0.15	0.22	0.66	51	0.0000882	2.29	0.0009889	34.0	0.0813766	32.3	0.075	0.012	0.282644	0.000007	0.282644	0.000007	-4.98	0.82
LCT-B z1b	0.553	0.020	0.639	0.022	1.00	0.54	0.752	1.10	0.17	0.38	0.43	38	0.0000858	3.70	0.0009858	54.0	0.0833311	51.3	0.072	0.010	0.282660	0.000004	0.282660	0.000004	-4.40	0.80
LCT-B z2b	0.550	0.024	0.636	0.025	0.67	0.64	0.753	0.72	0.11	0.34	0.32	36	0.0000853	4.32	0.0006624	94.8	0.0563347	91.6	0.069	0.009	0.282646	0.000004	0.282646	0.000004	-4.89	0.80
LCT-B z3b	0.5412	0.0022	0.6273	0.0089	0.69	0.06	0.743	0.57	0.85	0.25	3.38	211	0.0000840	0.42	0.0006755	8.5	0.0583727	8.2	0.135	0.016	0.282644	0.000003	0.282644	0.000003	-4.99	0.79
LCT-B z4b	0.559	0.010	0.645	0.013	0.62	0.26	0.762	0.50	0.18	0.23	0.78	64	0.0000868	1.79	0.0006156	41.2	0.0514731	39.9	0.056	0.007	0.282633	0.000004	0.282633	0.000004	-5.36	0.79
LCT-B z5b	0.5710	0.0086	0.657	0.012	0.60	0.23	0.752	0.74	0.37	0.39	0.94	70	0.0000886	1.51	0.0005885	38.1	0.0482044	37.0	0.084	0.012	0.282615	0.000006	0.282615	0.000006	-6.00	0.81
MFT-1 z1	1.225	0.018	1.311	0.020	1.48	0.46	0.765	0.72	0.17	0.17	1.02	72	0.0001901	1.49	0.0014554	31.1	0.0555597	29.9	0.057	0.008	0.282658	0.000005	0.282658	0.000005	-4.45	0.80
MFT-1 z2	1.223	0.020	1.309	0.021	1.59	0.50	0.762	0.87	0.12	0.12	0.97	67	0.0001898	1.60	0.0015674	31.3	0.0599280	30.1	0.065	0.009	0.282675	0.000005	0.282675	0.000005	-3.87	0.80
MFT-1 z3	1.241	0.015	1.327	0.017	1.58	0.37	0.762	0.85	0.11	0.08	1.32	85	0.0001926	1.20	0.0015569	23.7	0.0586604	22.8	0.081	0.012	0.282636	0.000006	0.282636	0.000006	-5.23	0.81
MFT-1 z4	1.227	0.021	1.313	0.023	1.37	0.54	0.763	0.78	0.13	0.15	0.88	64	0.0001904	1.73	0.0013490	39.4	0.0514216	38.1	0.084	0.013	0.282630	0.000008	0.282630	0.000008	-5.47	0.83
MFT-1 z5	1.240	0.019	1.326	0.021	1.50	0.30	0.776	1.05	0.18	0.29	0.61	49	0.0001923	1.56	0.0014791	20.3	0.0558016	19.1	0.074	0.011	0.282675	0.000006	0.282675	0.000006	-3.87	0.81
MFT-1 z6	1.179	0.047	1.265	0.048	1.12	0.94	0.781	0.73	0.04	0.14	0.31	33	0.0001829	3.99	0.0011042	83.9	0.0438053	80.8	0.072	0.012	0.282665	0.000010	0.282665	0.000010	-4.22	0.86
MFT-1 z7	1.208	0.044	1.294	0.045	1.87	1.15	0.757	0.95	0.19	0.44	0.42	39	0.0001873	3.63	0.0018417	61.4	0.0713279	58.7	0.079	0.012	0.282673	0.000005	0.282673	0.000005	-3.95	0.80
MFT-1 z8	1.204	0.049	1.290	0.050	2.06	1.28	0.756	1.07	0.12	0.31	0.39	36	0.0001868	4.07	0.0020328	62.2	0.0789491	59.2	0.077	0.010	0.282696	0.000005	0.282696	0.000005	-3.12	0.80
MFT-1 z9	1.217	0.032	1.303	0.034	1.47	0.83	0.770	0.83	0.10	0.17	0.58	48	0.0001887	2.67	0.0014474	56.3	0.0556415	54.3	0.064	0.009	0.282680	0.000009	0.282680	0.000009	-3.69	0.84
MFT-1 z10	1.198	0.024	1.284	0.025	1.34	0.62	0.759	0.97	0.14	0.18	0.78	57	0.0001858	2.00	0.0013245	45.8	0.0517295	44.3	0.081	0.011	0.282685	0.000004	0.282685	0.000004	-3.51	0.80
MFT-1 z11	1.209	0.020	1.295	0.021	1.22	0.48	0.779	0.70	0.12	0.12	0.99	71	0.0001876	1.61	0.0012016	39.5	0.0464803	38.3	0.085	0.013	0.282664	0.000007	0.282664	0.000007	-4.24	0.82
MFT-1 z12	1.230	0.032	1.316	0.033	2.09	0.83	0.757	1.05	0.10	0.17	0.61	47	0.0001908	2.60	0.0020562	40.0	0.0781938	38.0	0.080	0.012	0.282668	0.000007	0.282668	0.000007	-4.11	0.82
MFT-1 z1b	1.168	0.045	1.254	0.046	0.75	0.70	0.773	0.43	0.12	0.57	0.21	31	0.0001812	3.83	0.0007376	93.4	0.0295334	90.5								
MFT-1 z2b	1.204	0.016	1.290	0.018	1.50	0.25	0.770	0.58	0.17	0.27	0.62	54	0.0001868	1.34	0.0014826	16.6	0.0575838	15.6								
MFT-1 z3b	1.229	0.021	1.315	0.023	1.28	0.33	0.779	0.86	0.13	0.24	0.54	47	0.0001906	1.72	0.0012632	25.7	0.0480815	24.3	0.089	0.014	0.282683	0.000010	0.282683	0.000010	-3.61	0.85
HRT-A z1	2.0255	0.0793	2.112	0.080	5.33	1.58	0.755	1.29	0.22	0.61	0.36	32	0.0003143	3.92	0.0052599	29.7	0.1214442	26.9	0.055	0.008	0.282569	0.000005	0.282569	0.000005	-7.60	0.80
HRT-A z2.1	1.9913	0.0144	2.077	0.017	2.29	0.36	0.758	0.77	0.43	0.20	2.15	131	0.0003089	0.72	0.0022559	15.6	0.0529826	15.1	0.068	0.009	0.282576	0.000005	0.282576	0.000005	-7.33	0.80
HRT-A z2.2	1.9947	0.0149	2.081	0.017	2.47	0.38	0.755	0.75	0.46	0.24	1.96	122	0.0003095	0.75	0.0024401	15.5	0.0572110	14.9	0.057	0.007	0.282547	0.000003	0.282547	0.000003	-8.35	0.79
HRT-A z3	1.9707	0.0296	2.057	0.031	2.24	0.77	0.758	0.48	0.14	0.15	0.90	69	0.0003058	1.50	0.0022053	34.4	0.0523338	33.3	0.053	0.008	0.282151	0.000006	0.282151	0.000006	-22.38	0.81
HRT-A z4	2.0067	0.0176	2.093	0.020	2.30	0.44	0.753	0.65	0.42	0.26	1.65	108	0.0003113	0.88	0.0022700	19.3	0.0529031	18.7	0.083	0.013	0.282581	0.000009	0.282581	0.000009	-7.17	0.84
HRT-A z5	2.1258	0.0681	2.212	0.069	3.77	1.79	0.752	0.82	0.30	0.67	0.46	41	0.0003298	3.20	0.0037225	47.5	0.0818915	45.1	0.069	0.011	0.282227	0.000007	0.282227	0.000007	-19.68	0.81
HRT-A z7	1.9784	0.0468	2.064	0.048	2.68	1.22	0.756	0.55	0.07	0.13	0.58	50	0.0003069	2.37	0.0026403	45.7	0.0624154	43.9	0.047	0.008	0.282146	0.000008	0.282146	0.000008	-22.54	0.83
HRT-A z9	1.9853	0.0677	2.071	0.068	2.89	1.68	0.771	0.45	0.18	0.42	0.43	42	0.0003080	3.41	0.0028496	58.3	0.0671283	55.7	0.050	0.008	0.282128	0.000006	0.282128	0.000006	-23.20	0.81
HRT-B z1	2.0081	0.0141	2.094	0.017	2.27	0.37	0.746	0.57	1.13	0.59	1.93	125	0.0003116	0.70	0.0022364	16.2	0.0520850	15.7	0.046	0.006	0.282551	0.000003	0.282551	0.000003	-8.23	0.79
HRT-B z3	1.9823	0.0145	2.068	0.017	2.21	0.37	0.753	0.73	0.44	0.22	2.00	125	0.0003076	0.73	0.0021772	16.7	0.0513669	16.1	0.060	0.009	0.282585	0.000005	0.282585	0.000005	-7.01	0.80
HRT-B z4	1.9969	0.0230	2.083	0.025	2.74	0.46	0.759	0.75	0.29	0.27	1.07	71	0.0003098	1.15	0.0026990	16.7	0.0632122	15.8	0.059	0.008	0.282593	0.000006	0.282593	0.000006	-6.76	0.81
HRT-B z5	2.0923	0.0195	2.178	0.021	2.51	0.49	0.748	0.79	0.74	0.46	1.61	102	0.0003246	0.93	0.0024785	19.4	0.0553997	18.8	0.068	0.010	0.282575	0.000008	0.282575	0.000008	-7.39	0.83
HRT-B z6	1.9988	0.0117	2.085	0.015	2.25	0.28	0.770	0.72	0.25	0.09	2.89	173	0.0003101	0.59	0.0022159	12.4	0.0518481	11.9	0.072	0.012	0.282591	0.000009	0.282591	0.000009	-6.81	0.84
HRT-B z7	1.9934	0.0128	2.079	0.015	2.31	0.30	0.750	0.65	0.51	0.20	2.49	153	0.0003093	0.64	0.0022780	13.0	0.0534461	12.5	0.057	0.009	0.282531	0.000007	0.282531	0.000007	-8.93	0.82
HRT-B z8	2.0073	0.0050	2.093	0.010	2.21	0.12	0.738	0.66	1.53	0.25	6.06	348	0.0003114	0.25	0.0021747	5.5	0.0506692	5.4	0.058	0.007	0.282602	0.000004	0.282602	0.000004	-6.43	0.79
HRT-B z9	1.9937	0.0095	2.080	0.013	2.20	0.24	0.752	0.62	0.82	0.28	2.98	182	0.0003093	0.48	0.0021689	11.1	0.0508756	10.7	0.057	0.008	0.282542	0.000003	0.282542	0.000003	-8.56	0.79
HRT-B z10	1.9796	0.0191	2.066	0.021	2.45	0.50	0.751	0.76	0.46	0.31	1.48	96	0.0003071	0.97	0.0024121	20.4	0.0569857	19.7	0.063	0.008	0.282601	0.000005	0.282601	0.000005	-6.45	0.80
HRT-B z11	2.0144	0.0115	2.100	0.014	2.36	0.30	0.751	0.68	0.80	0.33	2.44	150	0.0003125	0.57	0.0023242	12.8	0.0539602	12.3	0.063	0.008	0.282588	0.000005	0.282587	0.000005	-6.94	0.80
HRT-B z1b	2.0278	0.0176	2.114	0.020	2.30	0.27	0.758	0.63	0.43	0.46	0.94	72	0.0003146	0.87	0.0022699	11.6	0.0523508	11.0	0.048	0.008	0.282627	0.000022	0.282626	0.000022	-5.56	1.09
HRT-B z2b	2.0013	0.0077	2.087	0.012	2.19	0.12	0.758	0.62	0.78	0.36	2.17	144	0.0003105	0.39	0.0021622	5.4	0.0505272	5.2	0.054	0.009	0.282569	0.000022	0.282569	0.000022	-7.59	0.94
HRT-B z3b	1.9920	0.0132	2.078	0.016	2.21	0.20	0.777	0.58	0.58	0.45	1.27	92	0.0003091	0.67	0.0021790	9.0	0.0511592	8.5	0.060	0.007	0.282583	0.000022	0.282583	0.000022	-7.08	0.79
HRT-B z4b	1.9806	0.0099	2.067	0.013	2.37	0.26	0.748	0.63	1.17	0.43	2.72	166	0.0003073	0.50	0.0023349	11.0	0.0551329	10.6	0.100	0.012	0.282554	0.000022	0.282554	0.000022	-8.11	0.80
HRT-B z5b	1.9945	0.0283	2.081	0.030	2.79	0.75	0.752	0.76	0.37	0.37	0.99	70	0.0003094	1.42	0.0027490	26.8	0.0644578	25.8	0.050	0.008	0.282530	0.000022	0.282530	0.000022	-8.98	0.82
HRT-C z1	1.9814	0.0207	2.067	0.022	2.17	0.41	0.803	0.45	0.19	0.15	1.24	85	0.0003074	1.04	0.0021370	18.8	0.0504407	18.0	0.041	0.005	0.282142	0.000004	0.282142	0.000004	-22.70	0.79
HRT-C z2	1.9867	0.0133	2.073	0.016	2.16	0.31	0.777	0.72	0.27	0.10	2.57	155	0.0003082	0.67	0.0021258	14.4	0.0500432	13.9	0.078	0.012	0.282591	0.000008	0.282591	0.000008	-6.82	0.82
HRT-C z3	1.9741	0.1084	2.060	0.109	3.49	2.86	0.752	0.67	0.16	0.64	0.25	31	0.0003063	5.49	0.0034432	82.2	0.0815704	78.2	0.059	0.009	0.282145	0.000006	0.282144	0.000006	-22.60	0.81
HRT-C z4	1.9954	0.0274	2.081	0.029	2.21	0.43	0.757	0.44	0.19	0.34	0.56	52	0.0003096	1.37	0.0021812	19.4	0.0511231	18.4	0.058	0.009	0.282137	0.000007	0.282137	0.000007	-22.88	0.82
HRT-C z5	1.9612	0.0396	2.047	0.040	1.96	0.89	0.791	0.46	0.17	0.20	0.83	65	0.0003043	2.02	0.0019320	45.7	0.0460723	44.1	0.067	0.010	0.282156	0.000009	0.282156	0.000009	-22.21	0.84
HRT-C z6	1.9896	0.0581	2.076	0.059	2.59	1.12	0.793	0.57	0.07	0.15	0.44	40	0.0003087	2.92	0.0025563	43.4	0.0600866	41.1	0.054	0.008	0.282140	0.000008	0.282140	0.000008	-22.75	0.83
HRT-C z7	47.7025	0.0212	47.789	0.023	179.80	0.19	0.636	0.79	7.13	0.11	65.78	3334	0.0074273	0.04	0.1937271	0.1	0.1892576	0.1	0.043	0.006	0.282167	0.000005	0.282167	0.000005	-21.79	0.80
HRT-C z8	1.9937	0.0191	2.080	0.021	2.44	0.49	0.757	0.50	0.17	0.12	1.45	100	0.0003093	0.96	0.0024082	20.0	0.0564925	19.2	0.052	0.008	0.282147	0.000007	0.282147	0.000007	-22.51	0.81
HRT-C z9	2.0163	0.0113	2.102	0.014	2.39	0.29	0.749	0.52	0.72	0.30	2.38	152	0.0003128	0.56	0.0023536	12.3	0.0545913	11.9	0.073	0.011	0.282158	0.000005	0.282158	0.000005	-22.14	0.80
HRT-C z10	1.9832	0.0239	2.069	0.025	2.29	0.58	0.771	0.51	0.12	0.10	1.26	89	0.0003077	1.21	0.0022565	25.3	0.0532107	24.4	0.048	0.007	0.282143	0.000007	0.282143	0.000007	-22.67	0.82
HRT-C z1b	2.0285	0.0332	2.115	0.034	2.65	0.52	0.768	0.49	0.17	0.34	0.50	48	0.0003147	1.64	0.0026094	19.8	0.0601592	18.5	0.059	0.010	0.282145	0.000022	0.282145	0.000022	-22.57	1.49
HRT-C z2b	2.0145	0.0287	2.101	0.030	2.38	0.45	0.780	0.46	0.19	0.33	0.58	53	0.0003125	1.43	0.0023482	19.1	0.0545162	18.0	0.062	0.010	0.282091	0.000022	0.282091	0.000022	-24.49	1.44
HRT-C z3b	2.0228	0.0429	2.109	0.044	2.71	0.67	0.775	0.52	0.20	0.52	0.39	41	0.0003138	2.12	0.0026696	24.6	0.0617206	23.0	0.049	0.008	0.282157	0.000022	0.282157	0.000022	-22.17	0.85
SRB-2 z1	2.1576	0.0266	2.244	0.028	3.00	0.70	0.751	0.85	0.74	0.63	1.17	78	0.0003348	1.23	0.0029552	23.4	0.0640546	22.5	0.087	0.013	0.282591	0.000009	0.282591	0.000009	-6.82	0.84
SRB-2 z2	2.0760	0.0128	2.162	0.015	2.53	0.32	0.750	0.67	0.53	0.22	2.35	145	0.0003221	0.62	0.0024930	12.9	0.0561627	12.4	0.052	0.008	0.282588	0.000006	0.282588	0.000006	-6.93	0.81
SRB-2 z3	2.0621	0.0226	2.148	0.024	2.37	0.53	0.763	0.70	0.17	0.11	1.50	98	0.0003199	1.10	0.0023412	22.5	0.0530966	21.7	0.081	0.013	0.282636	0.000007	0.282636	0.000007	-5.22	0.82
SRB-2 z4	2.0547	0.0231	2.141	0.025	2.55	0.60	0.755	0.67	0.44	0.35	1.25	86	0.0003188	1.13	0.0025174	23.5	0.0572989	22.7	0.059	0.008	0.282514	0.000004	0.282514	0.000004	-9.55	0.79
SRB-2 z5	2.0597	0.0085	2.146	0.012	2.13	0.21	0.742	0.74	0.45	0.12	3.79	220	0.0003196	0.41	0.0020952	9.8	0.0475744	9.5	0.092	0.013	0.282610	0.000005	0.282610	0.000005	-6.13	0.80
SRB-2 z6	2.0702	0.0129	2.156	0.016	2.24	0.31	0.769	0.64	0.31	0.12	2.50	155	0.0003212	0.62	0.0022082	13.9	0.0498853	13.5	0.077	0.012	0.282602	0.000009	0.282602	0.000009	-6.44	0.85
SRB-2 z7	2.0563	0.0280	2.142	0.029	2.56	0.73	0.754	0.71	0.76	0.74	1.03	73	0.0003190	1.36	0.0025202	28.8	0.0573164	27.8	0.062	0.009	0.282549	0.000004	0.282549	0.000004	-8.31	0.79
SRB-2 z8	2.0700	0.0102	2.156	0.013	2.18	0.16	0.772	0.90	0.58	0.31	1.85	119	0.0003212	0.49	0.0021528	7.2	0.0486376	6.8	0.054	0.007	0.282559	0.000004	0.282559	0.000004	-7.95	0.79
SRB-2 z9	2.0582	0.0076	2.144	0.011	2.10	0.19	0.751	0.61	0.54	0.14	3.98	239	0.0003193	0.37	0.0020660	9.0	0.0469436	8.7	0.081	0.011	0.282580	0.000005	0.282580	0.000005	-7.21	0.80
SRB-2 z10	2.1227	0.0077	2.209	0.012	2.31	0.19	0.752	0.76	0.53	0.13	4.23	243	0.0003293	0.36	0.0022771	8.3	0.0501683	8.1	0.070	0.010	0.282624	0.000004	0.282624	0.000004	-5.65	0.79
SRB-2 z1b	2.0674	0.0143	2.153	0.017	2.02	0.22	0.784	0.63	0.31	0.24	1.28	93	0.0003208	0.69	0.0019887	10.8	0.0449862	10.3								
SRB-2 z5b	2.1117	0.0223	2.198	0.024	2.32	0.35	0.768	0.79	0.28	0.34	0.82	64	0.0003276	1.06	0.0022909	15.1	0.0507368	14.3								
TEM-1																			0.046	0.007	0.282693	0.000016	0.282685	0.000016	5.77	0.97
TEM-2																			0.056	0.009	0.282698	0.000014	0.282688	0.000014	5.85	0.93
TEM-3																			0.047	0.007	0.282710	0.000010	0.282702	0.000010	6.36	0.85
TEM-4																			0.049	0.007	0.282689	0.000008	0.282681	0.000008	5.61	0.83
TEM-5																			0.047	0.007	0.282721	0.000009	0.282713	0.000009	6.75	0.84
TEM-6																			0.050	0.007	0.282694	0.000008	0.282686	0.000008	5.80	0.83
TEM-7																			0.051	0.007	0.282692	0.000007	0.282684	0.000007	5.72	0.82
TEM-8																			0.051	0.008	0.282695	0.000008	0.282687	0.000008	5.82	0.83
TEM-9																			0.052	0.007	0.282695	0.000009	0.282687	0.000009	5.82	0.85
TEM-10																			0.048	0.007	0.282675	0.000007	0.282667	0.000007	5.12	0.82
TEM-11																			0.041	0.007	0.282703	0.000006	0.282695	0.000006	6.12	0.81
TEM-12																			0.041	0.007	0.282692	0.000008	0.282684	0.000008	5.74	0.83
TEM-13																			0.040	0.007	0.282691	0.000007	0.282684	0.000007	5.73	0.82
TEM-14																			0.040	0.007	0.282702	0.000006	0.282695	0.000006	6.10	0.81
TEM-15																			0.056	0.009	0.282715	0.000013	0.282706	0.000013	6.48	0.91
TEM-16																			0.056	0.009	0.282730	0.000016	0.282720	0.000016	7.00	0.96
TEM-17																			0.040	0.007	0.282686	0.000007	0.282679	0.000007	5.55	0.82
TEM-18																			0.049	0.008	0.282679	0.000007	0.282671	0.000007	5.26	0.81
PLE-1																			0.010	0.001	0.282469	0.000008	0.282468	0.000008	-3.69	0.82
PLE-2																			0.010	0.001	0.282481	0.000008	0.282480	0.000008	-3.26	0.83
PLE-3																			0.010	0.001	0.282480	0.000008	0.282479	0.000008	-3.29	0.83
PLE-4																			0.010	0.001	0.282473	0.000009	0.282472	0.000009	-3.56	0.85
PLE-5																			0.010	0.001	0.282487	0.000007	0.282486	0.000007	-3.05	0.82
PLE-6																			0.273	0.001	0.282510	0.000012	0.282509	0.000012	-2.22	0.90
PLE-7																			0.273	0.001	0.282474	0.000011	0.282473	0.000011	-3.51	0.88
PLE-8																			0.010	0.001	0.282487	0.000006	0.282486	0.000006	-3.05	0.81
PLE-9																			0.010	0.001	0.282474	0.000006	0.282473	0.000006	-3.51	0.81
PLE-10																			0.010	0.001	0.282459	0.000006	0.282458	0.000006	-4.05	0.83

a Isotopic dates calculated using the decay constants λ(238) = 1.55125E-10 and λ(235) = 9.8485E-10 (ref. 62).
b Corrected for initial Th/U disequilibrium using the average Th/U partition coefficient ratio derived from melt inclusion analyses and published data (Figure S5).
d Th contents calculated from radiogenic $^{208}Pb/^{206}Pb$ assuming concordance between U-Th and Pb systems.
e Total mass of radiogenic Pb.
f Total mass of common Pb.
g Ratio of radiogenic Pb (including ^{208}Pb) to common Pb.
h Th/U ratio of magma from which mineral crystallized.
i Measured ratio corrected for fractionation and spike contribution only.
j Measured ratios corrected for fractionation, tracer, blank and initial Pb. Blank was corrected using the average composition of total procedural blank (TPB; average=0.17 ± 0.09 pg; n=17) measurements peformed throughout the course of this study: 206Pb/204Pb=18.102 ± 0.378, 207Pb/204Pb=15.352 ± 0.469; 208Pb/204Pb=38.01 ± 1.14 (all uncertainties are 1 S.D.). Common Pb in excess of blank was corrected using a composition computed from the model of ref. 61.
